# Simulated Dopamine Modulation of a Neurorobotic Model of the Basal Ganglia

**DOI:** 10.3390/biomimetics9030139

**Published:** 2024-02-25

**Authors:** Tony J. Prescott, Fernando M. Montes González, Kevin Gurney, Mark D. Humphries, Peter Redgrave

**Affiliations:** 1 Department of Computer Science, University of Sheffield, Sheffield S10 2TN, UK; 2 Departamento de Inteligencia Artificial, Universidad Veracruzana, Veracruz 91090, Mexico; fmontes@uv.mx; 3 Department of Psychology, University of Sheffield, Sheffield S10 2TN, UK; k.gurney@sheffield.ac.uk (K.G.); p.redgrave@sheffield.ac.uk (P.R.); 4 School of Psychology, University of Nottingham, Nottingham NG7 2RD, UK; mark.humphries@nottingham.ac.uk

**Keywords:** basal ganglia, dopamine, robot, Parkinson’s disease, dopamine dysregulation, neurorobotics, computational neuroscience, computational psychiatry

## Abstract

The vertebrate basal ganglia play an important role in action selection—the resolution of conflicts between alternative motor programs. The effective operation of basal ganglia circuitry is also known to rely on appropriate levels of the neurotransmitter dopamine. We investigated reducing or increasing the tonic level of simulated dopamine in a prior model of the basal ganglia integrated into a robot control architecture engaged in a foraging task inspired by animal behaviour. The main findings were that progressive reductions in the levels of simulated dopamine caused slowed behaviour and, at low levels, an inability to initiate movement. These states were partially relieved by increased salience levels (stronger sensory/motivational input). Conversely, increased simulated dopamine caused distortion of the robot’s motor acts through partially expressed motor activity relating to losing actions. This could also lead to an increased frequency of behaviour switching. Levels of simulated dopamine that were either significantly lower or higher than baseline could cause a loss of behavioural integration, sometimes leaving the robot in a ‘behavioral trap’. That some analogous traits are observed in animals and humans affected by dopamine dysregulation suggests that robotic models could prove useful in understanding the role of dopamine neurotransmission in basal ganglia function and dysfunction.

## 1. Introduction

The vertebrate basal ganglia are thought to play an important role in action selection—the resolution of conflicts between alternative motor programs [1,2,3,4,5,6,7]. The effective operation of basal ganglia circuitry and its regulation of motor behaviour are also known to rely on appropriate levels of the neurotransmitter dopamine (DA) [3,8,9]. For instance, dopamine antagonists (inhibitors), or dopamine-depleting brain lesions, have been found to impair a range of instrumental and spontaneous behaviours [10,11,12,13,14,15,16], affect the maintenance of behaviour over time [10,17], impair the initiation of movement [18,19], reduce behaviour switching [13,20,21,22,23], and can induce bradykinesia (slowed movement) or akinesia (lack of movement) [24,25]. Conversely, dopamine agonists (promoters) have been shown to cause increases in behaviour switching [21,22,26], or to lead to patterns of repetitive behaviour (stereotypy) [27,28,29,30]. Human basal ganglia-related disorders such as Parkinson’s disease (PD), schizophrenia, attention deficit hyperactivity disorder (ADHD), and Tourette’s syndrome are also known to involve abnormalities in the dopamine regulation of basal ganglia circuitry [31,32,33,34,35]. Nevertheless, in both humans and animals, there is still much to understand about how variation in tonic dopamine levels can have these different and variable effects on behaviour.

In this article, we show that when the tonic level of simulated dopamine in a robot model of the basal ganglia [36] is significantly reduced or increased, relative to a baseline, a variety of behavioural outcomes are observed that provide interesting comparisons with the results of animal studies, and with some of the observed behavioural consequences of dopamine dysregulation in disorders affecting the human basal ganglia. In this way, we hope that this article can contribute to the emerging field of computational psychiatry [37] and to the investigation of models of psychopathology via robotics [38].

The structure of the article is organised as follows. Section 2 describes some principles, derived from the study of animal behaviour, that allow us to measure the effectiveness of action selection. This section also provides an outline of the computational model of the vertebrate basal ganglia, viewed as an action selection mechanism, developed by Gurney, Prescott, and Redgrave [39,40] and extended by Humphries and Gurney [41]. This section also summarises the embedding of this model in the control architecture of a mobile robot, as previously reported by Prescott, Montes González et al. [36]. Section 3 describes Study 1, which concerns experiments with a non-embodied version of the model that provides fresh insights into the effects of tonic dopamine modulation on selection. Section 4 then describes Study 2, which applies ethological methods to analyse the results of experiments with the robot embedding of the model in which we vary the simulated level of tonic dopamine. Finally, Section 5 draws some comparisons with animal and human data, and discusses some of the implications of our study for the use of robotic modelling in neuroscience.

## 2. A Robot Model of Action Selection by the Basal Ganglia

### 2.1. Requirements for Effective Selection

The requirements for effective action selection in animal nervous systems have been previously analysed by a number of authors from the perspective of understanding how natural selection pressures could lead to the emergence of different action selection strategies and mechanisms [3,4,42,43,44,45]. In particular, we have previously argued that the need to provide fast and clean selection between alternative courses of action, and to do so efficiently with respect to computational and connectivity costs, has favoured the development of specialised action selection mechanisms, of which the vertebrate basal ganglia are an important example [3]. Here, we provided a summary of the key requirements; for further explanation and justification, see [3,44].

Given a set of competing and incompatible programs, the requirements for an effective action selection mechanism can be summarised as follows: (i) In selecting a winner, all else being equal, prefer the most strongly supported, or most salient, competitor as indicated by relevant external and internal cues. (ii) Allow only one program to be expressed at a given time; this winner should be cleanly selected (i.e., allowed unrestricted access to the motor apparatus), and the losers should be prevented from interfering with its performance, termed lack of distortion. (iii) Provide clean switching—a competitor with a slight edge over its rivals should see the competition resolved rapidly and decisively in its favour. (iv) Support action maintenance—a winning competitor may be required to remain active at lower salience levels than are initially required for it to overcome the competition. This latter characteristic, also termed hysteresis [46] or behavioural persistence [47], can prevent unnecessary switching, or ‘dithering’, between closely matched competitors.

Note that this view of action selection treats input salience as a ‘common currency’ [3,48], in accordance with which diverse behavioural options can be evaluated for possible selection. The selector does not need to know what the option is, only how salient it is, with salience being determined by genetics and learning. We note that other ways of selecting between actions are possible that do not rely on salience computation, and that may well exist in the brain. These could operate in a complementary fashion with the centralised action selection mechanisms considered here (see [45] for further discussion).

### 2.2. A Model of Basal Ganglia Intrinsic Circuitry

**Figure 1:**
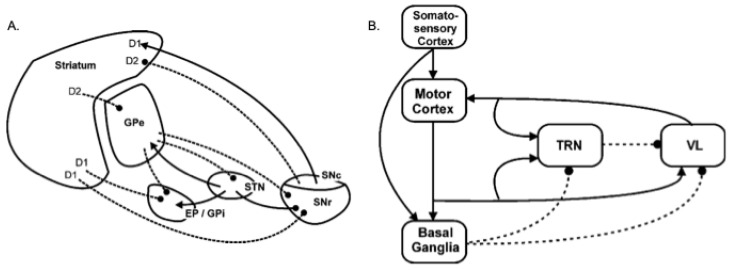
(**A**) A diagram of the connectivity, relative position, and relative size of the nuclei that comprise the vertebrate basal ganglia showing the separate projection targets of the D1 and D2 receptor striatal neurons as modelled by Gurney et al. [39,40]. (**B**) The connection scheme of the extended basal ganglia model, as modelled by Humphries and Gurney [41], incorporating a feedback pathway to the cortex via the thalamus. The box labelled ‘basal ganglia’ contains the functional anatomy shown on the left. Solid lines depict the excitatory pathway, and dotted lines depict inhibitory pathways in both diagrams. Anatomical labels are for the primate brain. Abbreviations: GPe—globus pallidus external segment; GPi—globus pallidus internal segment (EP—entopeduncular nucleus in rat); STN—subthalamic nucleus; SNc—substantia nigra pars compacta; SNr—substantia nigra pars reticulata. TRN—thalamic reticular nucleus—VL—ventrolateral thalamus. Reprinted with permission from Ref. [41]. 2002, Taylor & Francis Informa UK Ltd—Journals.

In a series of computational models, Gurney and co-workers [39,40,41] showed that the intrinsic connectivity of the basal ganglia, shown in Figure 1 (left), can meet many of these requirements for effective selection via a variety of mechanisms centred on the following: (i) a set of pathways from the striatum, the basal ganglia’s chief input nucleus, that can generate focused inhibition in basal ganglia output structures—the substantia nigra pars reticulata (SNR) and the globus pallidus internal segment (GPi) (entopeduncular nucleus in rats) [2]; (ii) diffuse excitation of these output structures by the subthalamic nucleus (STN) [49]; and (iii) regulation of the contrast between this focused striatal inhibition and diffuse STN excitation by the globus pallidus external segment (GPe) [39,50,51]. The overall mechanism, which is consistent with several theoretical accounts (e.g., [2,3,6]), is one that selects by removing tonic inhibition of motor pathways provided by basal ganglia outputs, for selected actions only, whilst maintaining or increasing inhibition of non-selected actions. The novelty of the Gurney et al. model included showing that intrinsic circuitry involving the GPe acts to regulate this selection effect, for instance, by normalising the level of surrounding inhibition for different numbers of competitors [40].

The balance between the different intrinsic basal ganglia mechanisms is also thought to depend on the level of tonic dopamine expression, which differentially impacts striatal projection neurons with different receptor types [39,52]. Specifically, striatal projection neurons can be separated into two broad classes. One population contains the neuropeptides substance P and dynorphin, preferentially expresses the D1 subtype of dopamine receptors, and projects directly to the output nuclei (SNr and GPi). Activity in these ‘D1 neurons’ suppresses the tonic firing in basal ganglia output structures, thus acting to select (disinhibit) target structures in the thalamus and brainstem [39,53]. A second population of projection neurons contains enkephalin and preferentially expresses D2 subtype dopamine receptors. The inhibitory projection from these ‘D2 neurons’ constitutes the first leg of an indirect, or control [39], pathway to the output nuclei that has two inhibitory links (Striatum–GPe; GPe–STN), followed by an excitatory one (STN–GPi/SNr). The net effect of D2 activity is therefore to activate output nuclei, increasing inhibitory control of the thalamus and brainstem [39,54,55]. Gurney et al. [40] demonstrated that simulation of increasing tonic dopamine in the model basal ganglia has the effect of increasing D1 neuron activity, reducing D2 activity, and consequently reducing activity in GPi/SNr. They concluded that raising tonic dopamine levels makes selection more ‘promiscuous’ increasing the likelihood that target motor pathways will be disinhibited, and potentially leading to ‘soft’ selection—the full or partial disinhibition of multiple channels.

### 2.3. A Model of the Extended Basal Ganglia

Humphries and Gurney [41] extended this intrinsic model, as shown in Figure 1 right, to include extrinsic feedback pathways via the ventral thalamus (VL) and the thalamic reticular nucleus (TRN). This new model provided improved selection, compared with that provide by the model of intrinsic circuitry alone, particularly with regard to generating clean selection with absence of distortion (the partial expression of losing channels) and the ability to maintain selected actions through positive feedback provided by the basal ganglia–thalamo-cortical loop. In Study 1 (Section 3), we present previously unpublished data and analysis obtained using this extended basal ganglia model, which casts light on how different tonic dopamine levels impact on its selection behaviour.

### 2.4. Robot Embedding of the Extended Basal Ganglia Model

**Figure 2:**
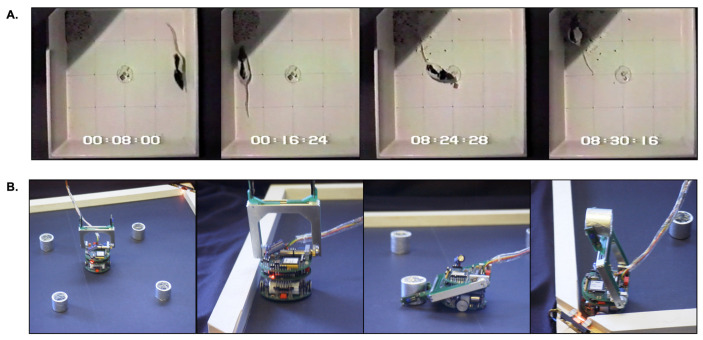
The model task. (**A**) A hungry rat placed in an open arena will initially explore the periphery (frames 1 and 2) before eventually venturing into the centre (frame 3) to retrieve food pellets that are then consumed in a sheltered ‘nest’ corner (frame 4). (**B**) In the robot, these behaviours are simulated by seeking (frame 1) and following walls (frame 2) and by searching for and acquiring cylinders (frame 3) that are then deposited in the lit corner of the arena (frame 4) (see Supplementary Video, part 2).

Prescott et al. [36,56] embedded the extended basal ganglia model [41] within the control architecture of a mobile robot in order to demonstrate that signal selection by the embedded model (as described for disembodied models above) could translate into effective action selection for an embodied agent expressing goal-directed behaviour. This model was based on consideration of the typical behaviour of a hungry rat placed in an open-topped arena with high sides (Figure 2A and Supplementary Video, part 1). In this situation, animals initially show fearful or thigmotaxic behaviour—avoiding open areas in the centre of the arena, whilst exploring walls and corners. As animals become more accustomed to the novel environment, they show foraging behaviour—collecting food pellets from a dish placed in the centre of the arena and typically consuming them in sheltered areas near the periphery. Salamone [10] showed that effective behaviour switching in a similar environment is compromised by the dopamine antagonist haloperidol and by dopamine-depleting lesions of the striatum. Hence, the task is an appropriate one for investigation of the effects of variation in simulated dopamine on robot action selection.

In the robot model of this task (Figure 2B and Supplementary Video, part 2), a table-top Khepera I mobile robot with a gripper turret is placed in a rectangular arena with illuminated corners to simulate safe places, and with small foil-covered cylinders to simulate food rewards. Fearful behaviour is simulated as staying close to walls and corners. Foraging involves searching for, locating, and picking up the cylinders. Consummatory behaviour is modelled as carrying a cylinder to one of the two illuminated corners and depositing it there. 

To generate appropriate behaviour, robot activity is decomposed into five *action sub-systems* inspired by the ethological classification of behaviour. Three of the five action sub-systems—*cylinder-seek*, *wall-seek*, and *wall-follow*—map patterns of input from the robot’s sensors into movements that orient the robot towards or away from specific types of stimuli (e.g., object contours). These behaviours can be viewed as belonging to the ethological category of orienting responses or taxes (e.g., see [57]). The two remaining sub-systems—*cylinder-pickup* and *cylinder-deposit*—generate carefully timed movement sequences that achieve specific behavioural outcomes and are modelled on the ethological concept of a fixed action pattern (FAP) [58]. Each action sub-system generates its preferred action at a given moment in the form of a motor vector that specifies target values for the speeds of the two wheels, and for the positions of the gripper arm (raised/lowered) and gripper jaw (open/shut). In the case of the orienting responses, the preferred action is computed using the sensory information available to the robot at that moment. In the case of FAPs, action specification can also depend on the current value of an internal clock.

In order to make appropriate action selection decisions, the robot needs information about relevant external and internal cues. Signals pertaining to external cues are computed by perceptual sub-systems from the raw sensory data available to the robot via an array of infra-red distance sensor signals, an ambient light sensor, and an optical sensor in the robot gripper. These sensory inputs are used to compute four bipolar signals indicating: the presence (+1) or absence (−1) of a nearby wall, nest area, or cylinder, or of an object in the robot gripper. Internal state cues are provided in the form of two real-valued intrinsic drives, loosely analogous to hunger and fear, as calculated by two motivational sub-systems. In the model, ‘fear’ is calculated as a function of exposure to the environment and is reduced with time spent in the environment, whilst ‘hunger’ gradually increases with time and is reduced when cylinders are deposited in the nest corners of the arena.

**Figure 3:**
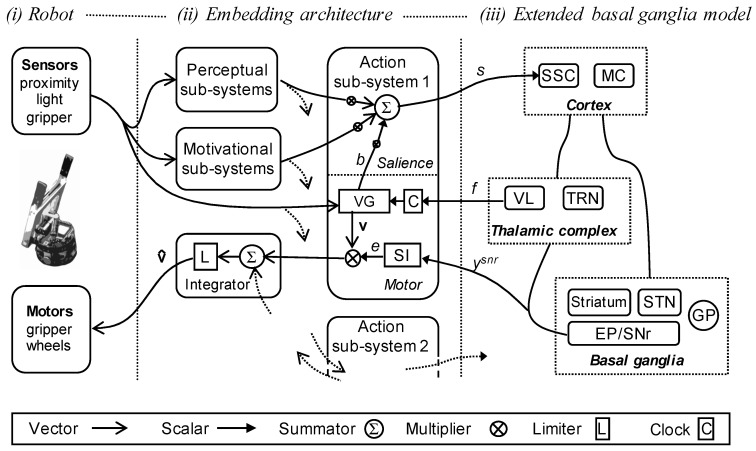
The robot basal ganglia model. The robot (i) interfaces, via the embedding architecture (ii), with the extended basal ganglia model (iii). The embedding architecture is composed of five action-subsystems (one shown), perceptual and motor sub-systems, and an integrator that combines the gated motor output of all five channels. See text, Section 4.2, the Supplementary Methods, and [36] for further explanation. Abbreviations: VG—(motor) vector generator; SI—shunting inhibition (Equation (1)); *e*—gating signal; *b*—busy signal; *s*—salience signal; *f*—feedback signal; $y^{snr}$—basal ganglia output; **v**—motor vector; $\hat{v}$—aggregate motor vector; SSC—somatosensory cortex; MC—motor cortex (other anatomical abbreviations as per Figure 1). Reprinted with permission from [36]. 2006, Elsevier Science and Engineering Journals.

Figure 3 shows how these different component sub-systems come together and interact with the embedded basal ganglia model. The model is composed of three parts: (i) the robot and its sensory and motor systems; (ii) the embedding architecture, that is, the set of perceptual, motivational, action sub-systems; and its interface to (iii) the extended basal ganglia model. Connections for the first of the five action sub-systems are shown; projections to and from the other action sub-systems are indicated by dotted lines.

As shown in Figure 3, each action sub-system takes inputs from the perceptual and motivational sub-systems, and from an internally generated busy signal (*b*) that is only non-zero if the action is currently selected, and that allows that sub-system to selectively boost its own salience. Based on these inputs, the action sub-system generates a weighted sum (the weights are hand-tuned) that is an estimate of its own instantaneous salience (*s*). This signal is then provided as an input to the embedded basal ganglia model. At the same time, the action-generating component of the sub-system calculates its preferred motor vector based on the robot’s sensor input and a feedback signal (*f*) from the component of the basal ganglia model corresponding to the ventrolateral thalamus (VL). This feedback signal is used to update or reset the clock (C) for the action system (in the case of a FAP), and to trigger the busy signal that contributes to its salience calculation. Full details of the implementation of this model are provided in [36] and also described in the Supplementary Methods.

In our earlier publications [36,56], we reported on the development of the robotic model and its behaviour for a fixed value of tonic dopamine transmission selected to provide effective action selection capabilities. In Study 2 (Section 4), we report previously unpublished data and analysis showing the behaviour of this model for a wide range of values of simulated dopamine values and explore the usefulness of the model for understanding the effects of variation in tonic dopamine in animals and humans.

## 3. Study 1: Tonic Dopamine Modulation in the Extended Basal Ganglia Model

Before presenting results for the robot model, it is useful to investigate the response of a non-embodied version of the extended basal ganglia model to changes in tonic dopamine modulation as this will provide a helpful yardstick for evaluating the embodied robotic version. This investigation will also help us to better understand any specific consequences due to embodiment when we come to examine the robotic model. This investigation also builds on prior studies of simulated tonic dopamine modulation [40,41] by providing a fine-grained analysis across the spectrum of possible simulated DA levels.

### 3.1. Methods

**Figure 4:**
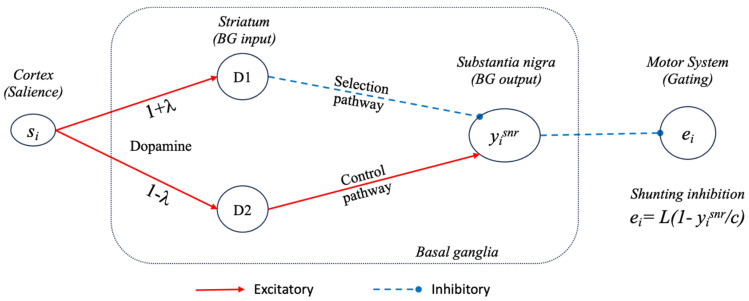
Processing within the *i*th basal ganglia channel. The salience of channel *i* is represented by the variable $s_{i}$. Leaky integrator units represent the activity in the input striatal units, with separate units for the D1- and D2-type neuron populations, and the substantia nigra output units. Other units within the model are not shown (see [36] and the Supplementary Methods). Synaptic efficacy is increased by tonic dopamine within the D1 channel (1 + λ) and reduced within the D2 channel (1 − λ). The basal ganglia output for channel *i* is modelled as affecting target motor systems via shunting inhibition (Equation (1)) and represented by the gating signal ($e_{i}$) for that channel.

Humphries and Gurney [41] provide a motivation for, and full description of, the extended basal ganglia model. Here, we note that this model, as well as the embedded version deployed in the robot, is based on standard ‘leaky integrator’ units, where one unit is used to represent activity in a pool of neurons in each of the modelled nuclei illustrated in Figure 1, and for each of the competing basal ganglia ‘channels’. As illustrated in Figure 3iii and Figure 4, input to channel *i* of the model, denoted as $s_{i}$, indicates the instantaneous salience of that channel, computed either by structures outside of the basal ganglia or by the striatal projection neurons themselves. The output for channel *i*, denoted as $y_{i}^{snr}$**,** indicates the instantaneous value of the inhibitory signal from the basal ganglia output nuclei to their targets elsewhere in the brain.

#### 3.1.1. Tonic Dopamine Modulation of the Model Basal Ganglia

Tonic dopamine modulation of the model is provided by a multiplicative factor in the equations, specifying afferent input to the striatum, the main input structure in the basal ganglia, based on a variable parameter, *λ*, where 0.0 *≤ λ ≤* 1.0. As illustrated in Figure 4, in striatal D1 units, where dopamine modulation increases synaptic efficacy, the effective weight is (1 + *λ*). In D2 units, where the effect is to reduce efficacy, the weight is (1 − *λ*). Note that the net effect of increasing dopamine is to increase inhibition on basal ganglia output structures via both the D1 and D2 internal pathways (labelled selection and control in Figure 4). Increasing inhibition of basal ganglia outputs in turn reduces basal ganglia inhibitory control of motor system targets, therefore making selection more promiscuous [39].

Previous studies have established that the basal ganglia model, in both its original [40] and extended forms [41], shows good selection properties, across a wide-range of salience pairings, with the simulated dopamine level set at around λ = 0.20. These studies also found an increasing prevalence of multiple-channel selection (see definition below) for λ values of 0.40 and above. Therefore, in the current analysis, we examined values of simulated dopamine ranging from 0.0 through to 0.5 in increments of 0.01. Note that the intention is to model changes in dopamine that happen over longer time scales and that we do not attempt, in this study, to model phasic short-latency dopamine responses that may also have an important effect on selection and that are considered to be play a critical role in some forms of learning [8,59].

#### 3.1.2. Using Basal Ganglia Outputs as Selection Signals

In order to consider the basal ganglia model as a model of selection, we need to interpret the effects of basal ganglia outputs on targets in the brainstem and thalamus. As noted above, selection corresponds to basal ganglia removing inhibition from the winner(s) and increasing inhibition on the losers. We assume that for any given channel, this effect varies between full disinhibition, partial inhibition, and full inhibition, and model this effect via a mechanism termed ‘shunting inhibition’, thought to capture some of the non-linear effects of the GABAergic outputs from basal ganglia on their targets in vivo (see [36]). Specifically, and as illustrated in Figure 4, for the *i*th channel, we define the selection, or *gating*, signal $e_{i}\;(0\;\leq \;e_{i}\leq 1)$ as follows:

(1) $$e_{i}=L\left(1-y_{i}^{snr}/c\right),\;$$

where *L*(*a*) is the piecewise linear function that forces $e_{i}\;$to lie between 0 and 1

(2) $$L(a)=\left\{\begin{array}{c} 0:a<0\\ a:0\leq a\leq 1,\\ 1:a>1 \end{array}\right.$$

Here, c is a constant equal to the value of $y_{i}^{snr}$ obtained when the basal ganglia model is run to convergence with zero-salience input on all channels (in other words, the tonic output level when there are no active competitors). The gating signal, $e_{i}$, is applied multiplicatively to adjust the gain of the *i*th channel. Thus, if $y_{i}^{snr}$ matches or exceeds the basal ganglia output when there are no active channels (which implies full inhibition of all channels since BG outputs are tonically active), then the effective gain is 0. On the other hand, if $y_{i}^{snr}$ falls below this level, due to positive-salience input in channel *i*, then $e_{i}$ will be non-zero and will be maximal when the basal ganglia outputs for channel *i* are fully inhibited ($y_{i}^{snr}=0).$ Modelling the effects of basal ganglia outputs using multiplicative gating builds on previous theoretical proposals that inhibitory synapses on or close to the cell body have a non-linear (multiplicative) effect [60,61] and on evidence from electron microscopy that GABAergic axon terminals from SNR to colliculus, in rats, are located on the soma and proximal dendrites of target neurons [62]. This interpretation of basal ganglia outputs as gating specific motor programs is also consistent with evidence showing that optogenetic activation of SNR cells, that oscillate in phase with rat consummatory behaviour, had the effect of inhibiting licking but did not affect other (non-consummatory) behaviours, such as those involving blinking and whisking, that are also controlled by the colliculus [63].

All parameters used were those described in Prescott et al. [36], and yielded a value of c=0.169 for Equation (1). For a detailed explanation of parameter setting in the wider model, see [36,40,41].

#### 3.1.3. Metrics for Measuring Effective Selection

Before progressing, it is useful to set out some criteria for evaluating the selection properties of the basal ganglia model for different levels of simulated dopamine. The gating signal, $e_{i}$, provides a normalised measure of selection *efficiency* that we can use to evaluate any given version of the model against our requirements for effective action selection (Section 2.1). It is useful to define some qualitative/categorical labels for different values of $e_{i}$. Allowing a 5% margin from absolute limits (based on common practice in statistical analyses of behaviour, and for ease of comparison with earlier studies [36,41]), we define the selection state of the ith competitor as *fully selected* if $0.95\leq e_{i}\leq 1.0$, *partially selected* if $0.05\leq e_{i}<0.95$, and *unselected* if $e_{i}<0.05$. It will also be useful to define specific metrics relating to the winning channel, *w*. Hence, we define $e_{w}={max}_{\forall i}e_{i}$ as the *efficiency* of the current winner, $1-e_{w}\;$as its *inefficiency*, and

(3) $$d_{w}=2\left(\sum_{i}e_{i}-e_{w}\right)/\sum_{i}e_{i}$$

as the level of *distortion* affecting the output of this winner. Note that $d_{w}$ will equal zero when all other competitors have zero efficiency, will increase with the number of partially disinhibited losers, and will be 1.0 or greater if two or more channels are fully disinhibited (multiple winners). Inspired by ethological research [64], we will also describe an uninterrupted series of time steps that share the same winner, and for which $e_{w}\geq 0.05$, as a single *bout* of behaviour.

Finally, we note that the result of the basal ganglia selection competition, as a whole, can be summarised by the vector **e**. It is useful to have some categorical labels to describe selection outcomes and in order to facilitate discussion of results. Following [36], and using the criteria just defined for single competitors, we assign the following qualitative labels to the possible outcomes of the full competition as defined by the instantaneous value of **e**:

*Clean selection*: One competitor fully selected; all others unselected.

*No selection*: All competitors unselected.

*Partial selection*: One or more competitors partially selected; no competitor fully selected.

*Distorted selection*: One competitor only fully selected; at least one other partially selected.

*Multiple selection*: Two or more competitors fully selected.

#### 3.1.4. Procedure

To better understand the effect of varying simulated dopamine on the selection properties of the extended basal ganglia model, we simulated a five-channel model, with two active channels, varying the salience, $s_{1}$, in channel 1 systematically from 0 to 1 in steps of 0.01, and then for each value of $s_{1}$, varying the salience, $s_{2}$, of channel 2 from 0 through to 1, again in steps of 0.01. For each resulting salience vector $\left(s_{1},\;s_{2},\;0,\;0,\;0\right)$, the model was run to convergence and the result classified in accordance with the scheme set out above. Importantly, selection competitions were run in sequence from low values to high values. The activations levels of all leaky integrator units in the model were initialised to zero for each new value of $s_{1}$ but thereafter, while that salience value was tested, were retained from one competition to the next. In other words, we simulated a situation where channel 1 was initially the only active channel, and gradually increased channel 2 while holding channel 1 constant. The goal here, is to simulate some aspects of the continuity of experience that we can expect in the robot model in which the recent history of selection competitions may influence the current competition through hysteresis.

### 3.2. Results

**Figure 5:**
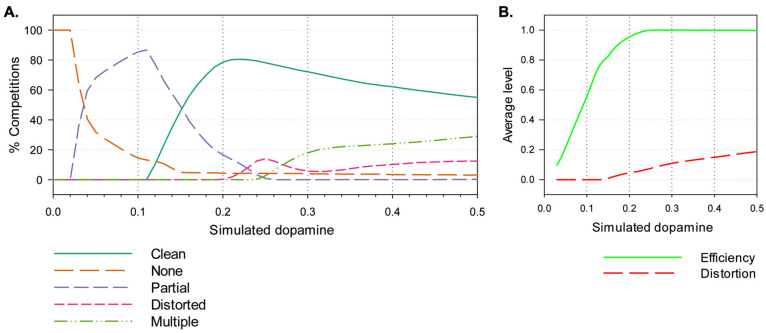
(**A**) The percentage of selection competitions falling into different classes of selection outcome for values of simulated dopamine, λ, ranging from 0 through to 0.5 in increments of 0.01. Data were obtained through an exhaustive search of a two-dimensional salience space. Partial selection is predominant for low dopamine values; distortion and multiple selection are evident at high dopamine values. Simulation with levels of λ > 0.5 resulted in continuation of the trends shown in the figure (see Supplementary Materials). (**B**) Average efficiency (green) and distortion (red) across all runs at each level of λ.

Figure 5A shows the percentage of action selection competitions, across the 500,000 (50 × 100 × 100) runs, falling into each of the selection classes—clean selection, no selection, partial selection, distortion, and multiple selection. Values of λ below 0.01 result in no selection, while for those in the range 0.04–0.15, partial selection predominates, and for those from 0.15 upwards, the majority of competitions end in clean selection with a peak around 0.22; distorted selection begins to appear with values above 0.2, and multiple selection occurs with levels of 0.25 and greater.

Figure 5B shows the average values of efficiency and distortion across all runs at a given level of λ. These graphs indicate that average efficiency increases, gradually reaching its maximal value (1.0) at λ = 0.23, while distortion increases gradually from zero beginning at around λ = 0.15 and reaching 0.2 by λ = 0.5.

**Figure 6:**
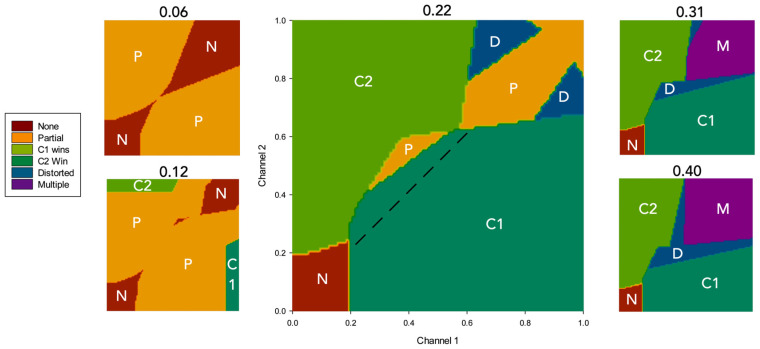
Selection boundaries in two-dimensional salience space for sample levels of simulated dopamine—very low (λ = 0.06), low (0.12), intermediate (0.22), high (0.31), and very high (0.40). For each plot, the salience of channel 1 is shown on the *x*-axis, and that of channel 2 is shown on the *y*-axis ranging from 0.0 to 1.0 (shown only for the central plot). Labels indicate the following: N—no selection; P—partial selection; C1—clean selection of channel 1; C2—clean selection of channel 2; D—distortion; M—multiple selection.

Figure 5 shows the average outcome at different levels of λ across all possible $\left(s_{1},\;s_{2}\right)$ dyads. In order to better understand the interplay between salience, simulated dopamine, and selection, Figure 6 shows the outcome of the simulation for five specific values of simulated dopamine (λ = 0.06, 0.12, 0.22, 0.31, and 0.40), indicating the boundaries of different classes of selection outcome on the $\left(s_{1},s_{2}\right)\;$plane. For clean selection only, the plots also distinguish between the selection of channel 1 (which is active first) and of channel 2 (which then competes for selection against channel 1).

Several properties of Figure 6 are worth noting. First, at all levels of λ, there is little or no selection at very low salience levels. This is largely a consequence of the threshold value of the model striatal input neurons, which serves to weed out weakly salient inputs. Second, with low λ (e.g., 0.12), clean selection (C1 or C2 in Figure 6) occurs, if at all, only when there is a high salience input in just one channel; otherwise, partial selection is the norm. Third, at all simulated dopamine levels, there is no clean selection for strong, evenly matched salience values (top-right corner of all plots). With low values of λ (0.06; 0.12), the outcome is no selection or partial selection of one or both channels, while with high values (0.31; 0.4), the result is distortion of the selected channel or multiple selection. The dotted line in the central plot (λ = 0.22) is shown to illustrate the extent of hysteresis in the model: channel 1 wins many selection competitions (encroaches across the diagonal) in which the salience of channel 2 is greater, purely because it is activated first.

**Figure 7:**
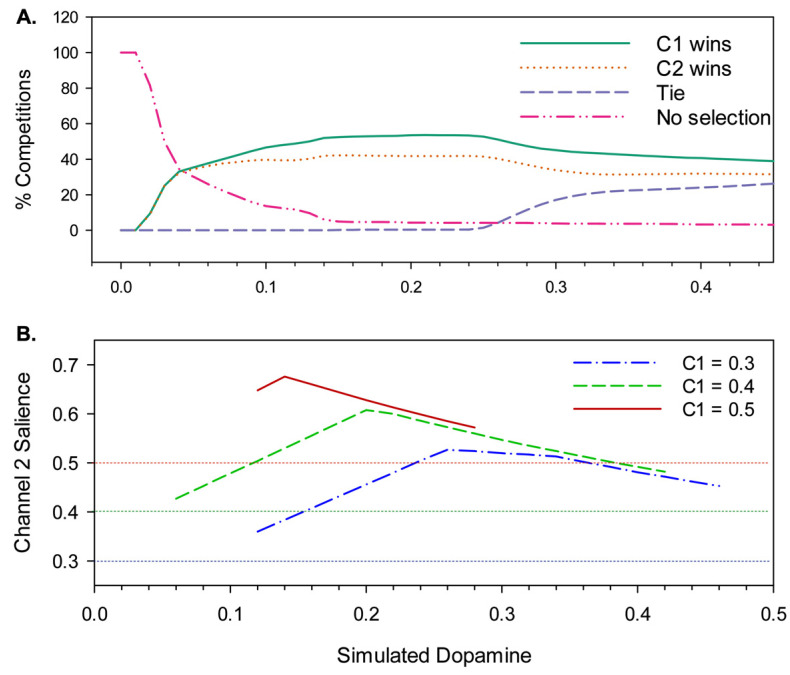
(**A**) Selection outcomes in the disembodied model re-classified as a channel 1 win, a channel 2 win, a stand-off (no selection), or a tie. Channel 1 (c1) wins substantially more competitions than channel 2 (c2) for all but the lowest levels of simulated dopamine. (**B**) The level of channel 2 salience, $s_{2}$, required for channel 2 to prevail (i.e., e2 > e1) against a channel 1 salience, $s_{1}$, of 0.3, 0.4, or 0.5, for different values of λ. Data are shown only where there is a clear switch from channel 1 to channel 2 with increasing $s_{2}$ (i.e., without an intervening interval of no selection or multiple selection). The degree of hysteresis varies depending on λ and $s_{1}$, with the value of λ that generates maximum hysteresis decreasing with increasing $s_{1}$.

To further our understanding of hysteresis in the model, the simulation results described above were reclassified to show the extent to which channel 1, which is always active first, is preferred to channel 2, irrespective of the selection outcome. Thus, the result of each competition was rescored as either a *channel 1 win* $\left(e_{1}>e_{2}\right)$, a *channel 2 win* ${(e}_{2}>e_{1})$, a *tie* ${(e}_{1}=e_{2}\neq 0)$, or *no selection* $\left(e_{1}=e_{2}=0\right)$. Figure 7A shows the results of this reclassification, and reveals that hysteresis is a property of the model for all but the lowest levels of simulated dopamine modulation (λ ≤ 0.06), with channel 1 consistently winning up to 10% more competitions than channel 2.

However, this is still not the full story. Figure 7B shows a further measure of hysteresis—the level of channel 2 salience required to overcome a given level of channel 1 salience—for three different initial fixed levels of $s_{1}$. The plot shows that hysteresis is governed by a complex interaction of λ with salience. Specifically, for values of $s_{1}$ in the range 0.3–0.5, the degree of hysteresis first increases with increasing λ, peaks, and then decreases; at its maximum, channel 2 salience needs to reach 176% of the channel 1 salience in order to win the selection competition. The peak λ value for hysteresis also changes for different values of $s_{1}$—as the salience of the selected channel increases, the value of λ at which hysteresis is maximal goes lower.

We conclude that the relatively flat level of hysteresis shown across a broad range of λ values in Figure 7A masks a significant dependency on salience. This outcome can be explained by understanding that hysteresis in the model occurs as a consequence of activity in the basal ganglia–thalamo-cortical feedback loop (via VL and TRN in Figure 1). Activity in this loop increases in proportion to reduced basal ganglia output; in other words, it increases with selection efficiency. With low values of λ, partial selection (low efficiency) predominates for low or intermediate salience values. This outcome results in less positive feedback via the thalamo-cortical pathway than for high-salience competitions. Consequently, when λ is low, hysteresis will be maximal with high salience. In contrast, high λ levels result in high-efficiency selection with comparatively low levels of salience input, thus generating substantial positive feedback and strong hysteresis. However, high-level salience competitions can result in the partial or full disinhibition of multiple channels (distorted or multiple selection). One consequence of this is an increase in TRN inhibition of the VL thalamus for the winning channel, resulting in a significant reduction in thalamocortical feedback for that channel. This means that with higher levels of λ, the current winner can be more vulnerable to interruption by its competitors.

## 4. Study 2: Selection in the Neurorobotic Basal Ganglia Model

Clean selection for the disembodied model, as illustrated in Figure 5, was above 75% in the simulated dopamine range 0.2 ≤ λ < 0.3, fell steeply to zero in the lower range 0.0 ≤ λ < 0.2, and fell more gradually (to 55%) in the higher range 0.3 ≤ λ ≤ 0.5. Defining these ranges as, respectively, *intermediate*, *low*, and *high* λ, and building on the analysis just described (and in earlier explorations in [36,40,41]), we can make the following hypotheses concerning the possible effects of varying simulated dopamine in the robotic model:

**Hypothesis** **1.**  *At intermediate levels of λ (0.2 ≤ λ < 0.3), we should expect to see a high proportion of clean selection with selected behaviours fully disinhibited and competing behaviours fully suppressed.*

**Hypothesis** **2.** *At low levels of λ (0.0 ≤ λ < 0.2), we should expect a predominance of partial selection or no selection (very low λ) and consequently the slowing or absence of movement.*

**Hypothesis** **3.** *For high levels of λ (0.3 ≤ λ), we should expect to see reduced inhibition of losing channels, leading to distorted or multiple selection, and resulting in motor commands that mix the movement requests of more than one action sub-system.*

**Hypothesis** **4.** *At both low and high levels of λ, we should expect to see changes in the hysteresis of selected channels modulated in accordance with the nature of the salience competition (e.g., whether the salience of competing channels is high, low, or evenly matched) as illustrated in Figure 7B. Changes to hysteresis can be expected to translate into consequences for action maintenance and for the timing of behavioural switching.*

With respect to each of these hypotheses, the observed behaviour of the robot may depend on a variety of factors related to its embodiment (discussed further below) and the requirement to generate sequences of integrated behaviour. Moreover, whereas the analysis in study 1 was based on an exhaustive search of an essentially two-dimensional salience space, the robot model samples behaviour-dependent trajectories through a five-dimensional salience space. The actual outcomes with respect to hypotheses 1–4 are therefore only partially predictable from the disembodied model and to be further determined from observation.

### 4.1. Methods

As illustrated in Figure 3ii and 4, for each action sub-system, *i*, the output of the basal ganglia, $y_{i}^{snr}$, is converted into a gating signal, $e_{i}$, via Equation (1), which is then used to scale the value of the motor vector for that action. An integrator module then sums up all of the motor vectors and passes the aggregate vector through a limiter (L) (Equation (2)) that constrains all values to lie in the range 0–1; this vector is then converted into the specific motor commands that control the robot.

Full details of the test environment, the robot sensor and motor systems, and the embedding architecture components, including their motivation in relation to the neuroscientific understanding of relevant brain sub-systems, are provided in [36], which also provides a broader discussion of the use of robotic models in neuroscience. Details of the full implementation of the robotic model and source code are provided in the Supplementary Materials. The basal ganglia model and robot embedding were implemented in C++, and the robot was controlled using Webots software (www.cyberbotics.com, accessed on the 23 February 2024) from a Linux workstation via an umbilical cable.

Note that the embedded basal ganglia model, which is simulated using the Euler method, is run to convergence for each time step of the robot model. The full robot model operates on a series of discrete time steps, providing sensor updates and modifying its action output at a rate of approximately 7 Hz; thus, it is always operating on the output of a fully converged model basal ganglia.

#### 4.1.1. Measuring Effective Action Selection in the Robot Model

**Figure 8:**
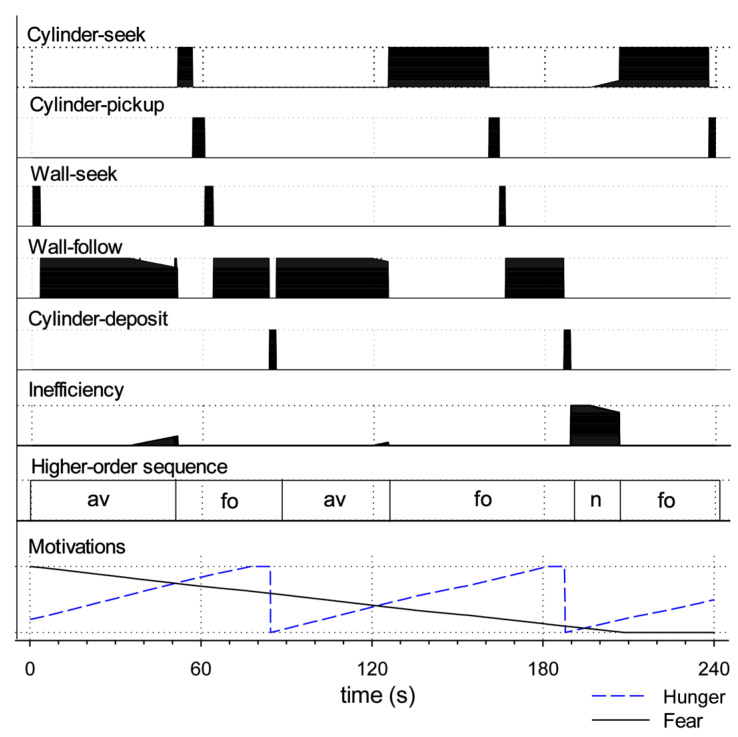
Bout/sequence structure of action selection in the robot model for a 240 s trial (λ = 0.20); the first 100 s is shown in the Supplementary Video, part 3. Each of the first five plots shows the efficiency (*e*) of selection for a given action sub-system plotted against time. The sixth plot shows the inefficiency of the current winner, the seventh the higher-order structure of the bout sequences, (av = avoidance; fo = foraging; n = no behaviour), and the final plot the levels of the two simulated motivations. All measures vary between 0 and 1 on the *y*-axis. The robot displays appropriate bouts of behaviour organised into integrated, goal-achieving sequences.

We will explore the results of our study using the methodology of the ethogram from behavioural science. This is illustrated in Figure 8 for a single 240 s run with simulated tonic dopamine set at an intermediate level of λ = 0.20. The top five lines of the plot show the value of the gating signal, $e_{i}$, for each of the five action sub-systems at each time step in the style of a behavioural ethogram. Comparing the different action sub-systems, it is evident that the robot generates extended sequences of behaviour with no more than one sub-system fully selected at any given time. The efficiency of selected actions is 100% or near it, actions are performed over extended bouts (solid blocks of high efficiency), and the inefficiency of the winner (plotted as the sixth line of the plot) is generally near-zero. In this run, the robot is initially fearful and seeks the wall (wall-seek), then switches into its wall-follow behaviour. This can be viewed as the robot forming higher-order sequence of avoidance (av) behaviour, as labelled in the seventh line of the plot. The final line of the plot shows the activity of the model motivational systems. As the level of simulated fear gradually subsides, simulated hunger increases. As a result, at around 50 s, the robot rapidly switches into its cylinder-seek behaviour. When it subsequently locates a cylinder, it switches to cylinder-pickup, then to wall-seek (this time carrying a cylinder), then wall-follow, and, when it finds a lit corner, cylinder-deposit. The higher-order action sequence beginning with cylinder-seek and ending with a successful deposit is labelled as foraging (fo) in the plot. Releasing the cylinder has the effect of reducing simulated hunger such that the robot is again motivated principally by fear to perform its avoidance-related behaviours (wall-seek and wall-follow). However, the level of simulated hunger gradually rises, which leads to two further higher-order foraging sequences interspersed by a period of no behaviour. The absence of behaviour occurs when neither of the intrinsic motivations is sufficiently strong to trigger any action—the robot sits idle, just as the rat might lie quietly in the corner of the arena.

From the perspective of the observer, the robot’s behaviour appears to be integrated and purposeful; individual action bouts are assembled into larger sequences that successfully reduce its drives. Below, we will compare this example of effective action selection and integrated behaviour with other runs in which the robot demonstrates various forms of behavioural disintegration as the result of lowering or raising the level of simulated dopamine in the model basal ganglia.

To further our analysis of behavioural (dis)integration, we have also developed a simple binary classification scheme to assess each trial according to its success in achieving higher-order behavioural goals. Specifically, we define ‘integrated behavior’ for this robotic task as constituting, at minimum, successful avoidance in the initial ‘high fear/low hunger’ phase, and a successful foraging sequence in the later ‘low fear/high hunger’ phase. Operationally, we define the following:

(i) *Successful avoidance* is activity resulting in the discovery of a wall (ignoring any cylinders encountered en route) followed by movement covering some distance along the wall’s length.
(ii) *Successful foraging* is activity resulting in the deposition of a cylinder in a ‘nest’ area.

This classification scheme proved simple enough to be applied during live observation of robot behaviour. In addition, automatic logs were recorded detailing the robot’s sensory, motivational, and basal ganglia state at each time step, and the bout structure of its behavioural selections, allowing us to reconstruct and analyse the robot’s behaviour post hoc.

#### 4.1.2. Procedure

Based on our analysis of the disembodied model, we decided to test the robot for 30 trials each at low, intermediate, and high simulated dopamine levels, with five trials, each lasting 120 s, at each of 18 different values of λ: low = 0.03, 0.06, 0.09, 0.12, 0.15, and 0.18; intermediate = 0.20, 0.21, 0.22, 0.23, 0.25, and 0.28; and high = 0.31, 0.34, 0.37, 0.40, 0.43, 0.46. This resulted in 90 trials in total. The robot started each trial in the centre of the arena, facing one of the four walls, with four cylinders placed 18 cm diagonally from each corner (Figure 2, right). Following our initial analysis, a further 26 trials were conducted using a quota sampling strategy, as explained in the Section 4.2 below. Finally, in order to better understand our results, we also performed an additional 90 trials in which we enforced ‘hard’ selection of the winning action, for comparison with the baseline model, which allows the simultaneous expression of multiple actions (‘soft’ selection).

Since the behaviour of the robot is susceptible to noise, we applied statistical methods (using the SPSS statistical package, vs. 28) to further analyse some results. In the statistical analyses reported below, we used an alpha value of 0.05 and report significance values as two-tailed. When comparing between conditions, we used Levene’s test to check whether or not samples had similar variance. Where this test is significant, we report “equal variances not assumed” and provide adjusted degrees of freedom and *p*-values.

### 4.2. Results

**Figure 9:**
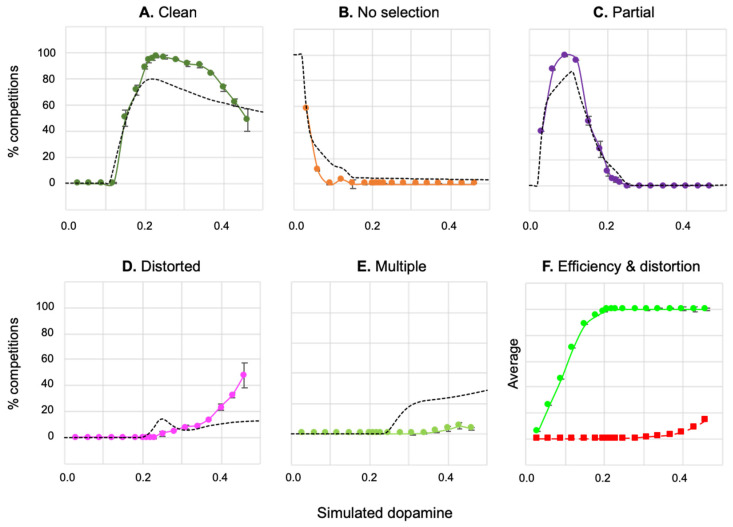
(**A**–**E**). The percentage of selection competitions falling into different classes of selection outcome for values of simulated dopamine ranging from 0.03 through to 0.46. Data were obtained by averaging five 120 s trials of robot behaviour, for each of the eighteen λ levels tested. Standard error bars are shown. Plots are coloured as per the colour scheme in Figure 5—clean selection (dark green), no selection (orange), partial selection (purple), distorted selection (pink), multiple selection (light green). Black dotted lines show the equivalent results obtained using the non-embodied model (Figure 5). Comparison of the selection properties of the non-embodied and robot models shows more clean, partial, and distorted selection in the robotic model and fewer selection competitions where the outcomes were either no selection or multiple selection. (**F**). Average efficiency (green) and distortion (red) across all runs at each level of λ.

In each trial, which typically consisted of around 800 robot time steps, the outcome of the basal ganglia selection competition, at each time step, was classified in accordance with the selection criteria specified in Section 3.1 above. For each λ value, the percentage of time steps resulting in each type of selection outcome was then averaged across all five trials regardless of the behavioural outcome of individual trials (which we consider next). The results of this analysis are shown in Figure 9A–E, for the initial 90 trials, together with a plot of average efficiency and distortion (as defined in Section 3.1) across the different λ levels (Figure 9F).

These results show the expected similarity between the selection profiles for the robotic and non-embodied models; nevertheless, there are some important differences. These include, in the robotic model, an increased proportion of partial selection at low λ levels (0.03 ≤ λ ≤ 0.12), of clean selection at intermediate and moderately high levels (0.2 ≤ λ ≤ 0.4), and of distorted selection at high levels (0.3 ≤ λ ≤ 0.46). There is also an almost complete absence of multiple selection at high λ levels. Whilst average efficiency is similar across the robotic and disembodied models, the robot model overall has less distortion except at the highest λ levels. In the intermediate range of simulated dopamine (λ = 0.20–0.29), clean selection for the robotic model is in the range of 89–95% compared to 73–81% for the disembodied model.

These results largely reflect the fact that the robot spends little time sampling the very-high-salience areas of the state space, or the very-low-salience areas, compared to the exhaustive search conducted for the disembodied model. This was confirmed via an analysis of salience values across 15 runs (one at each level of λ), which found that 95% of selection competitions were in the range of 0.3–0.75 for the winning channel and 0.2–0.7 for the strongest losing channel (see also [36] for a plot of how the salience space is sampled by the robot model). Note that that there may also be up to five channels with non-zero salience at any time as opposed to just two in the disembodied model. We next explore how the different levels of λ impacted on robot behaviour.

#### 4.2.1. Effects of Simulated Dopamine Modulation on Behavioural Outcome

The outcome of our initial binary analysis (see Section 4.1.1) was as follows. Seven levels of simulated dopamine (0.20–0.28 and 0.37) were scored as generating successful behaviour in all five trials; five levels (0.03–0.12 and 0.46) were unsuccessful in all trials, and the remaining six levels (0.15, 0.18, 0.31, 0.34, 0.40, and 0.43) generated a mixture of successful and unsuccessful trials.

**Figure 10:**
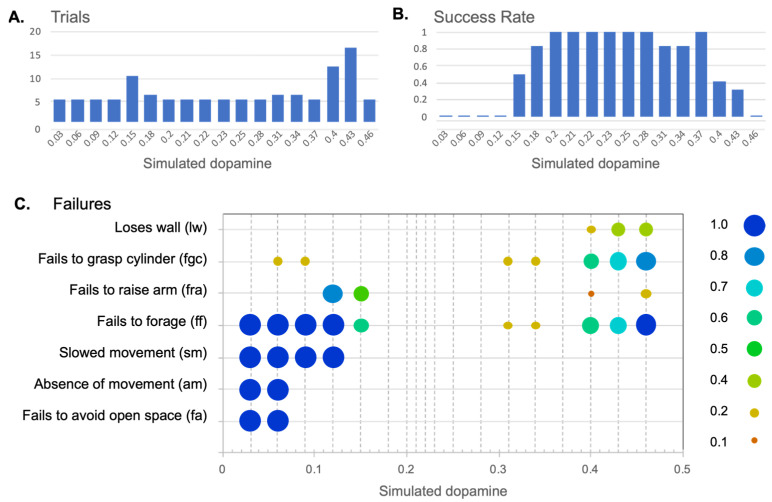
Total trials (**A**) and success rate ((**B**), 0.0–1.0) in achieving avoidance/foraging different levels of simulated dopamine (λ). (**C**) Evidence of disintegrated behaviour at different levels of λ. The bubble plot shows the proportion of trials at each value of λ that resulted in the observed failure type. See the text for further details.

In order to better understand what was happening at levels of λ that generated mixed results, a quota sampling strategy was implemented in which further trials were conducted until five successful trials in total, at each of these levels, had been achieved. This required between 1 and 11 trials per level, resulting in an additional 26 trials. Figure 10 shows the total trials (Figure 10A), and the overall success rate (Figure 10B) at different levels of λ, across all 116 trials, assessed against the criteria of success in both avoidance and foraging. Figure 10C shows a more detailed analysis of types of failures under the low- and high-λ regimes that we describe further below.

Figure 10B confirms that in the range of intermediate λ values (0.2–0.28), which generates high proportions of clean selection, as shown in Figure 9, the robot also reliably generates integrated sequences of behaviour. The absence of any failures in the 30 trials in this range provides a 95% level of confidence that the failure rate for this class of models is 10% or less.

**Figure 11:**
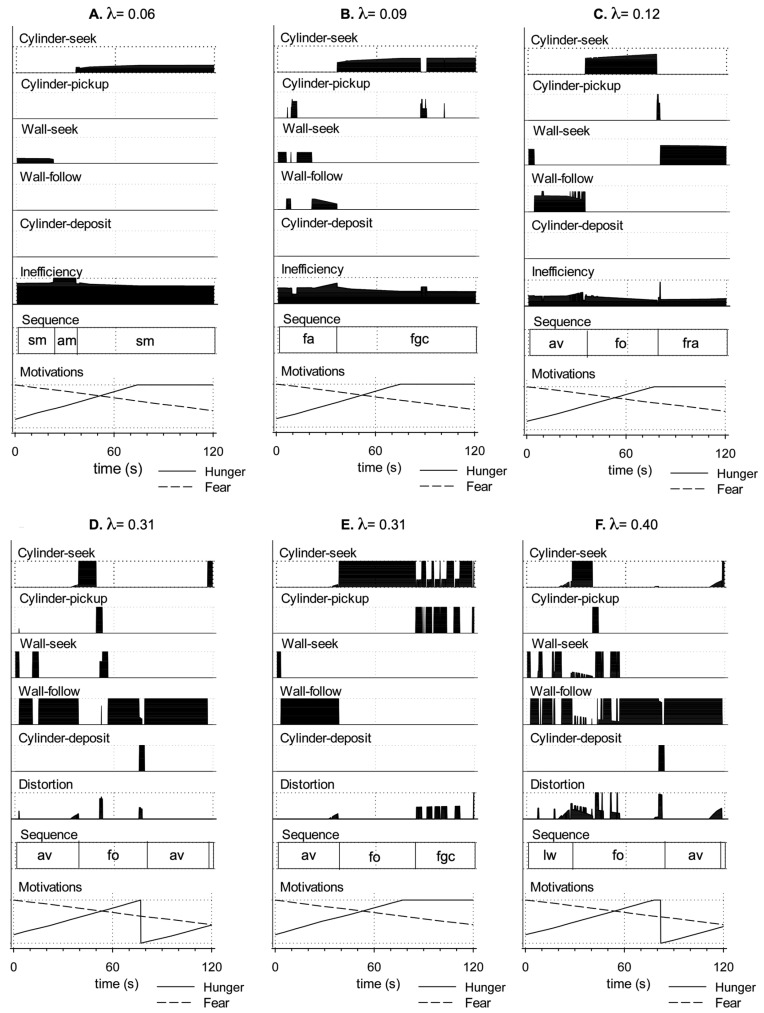
Bout/sequence structure of action selection in the robot model for three 120 s trials with low simulated dopamine, (**A**) λ = 0.06, (**B**) λ = 0.09, and (**C**) λ = 0.12, and three 120 s trials with high simulated dopamine: (**D**) λ = 0.31; (**E**) λ = 0.31; (**F**) λ = 0.40. The graph layout is as described for Figure 8, except that distortion, $d_{w}$, of the winning action, replaces inefficiency for panels D–F (as inefficiency is always zero in these trials). Labels in the ‘sequence’ plot show successful avoidance (av), foraging (fo), or different forms of behavioural disintegration as per Table 1. With low simulated dopamine, the robot shows slowed movement (sm) and an absence of movement (am). Inefficient selection can also cause premature deselection, leading to the failures to grasp the cylinder (fgc) or raise the gripper arm (fra) shown in plots B and C. With high values of λ, distortion of the selected behaviour by the motor output of losing competitors becomes a significant issue. Distortion in the run shown in plot D has only benign effects, but in the run shown in plot E causes behavioural disintegration as the robot fails to grasp a cylinder (fgc) despite multiple attempts. The run shown in plot F demonstrates that there is a high frequency of behaviour switching with high levels of simulated dopamine, in this case because distortion causes to the robot to repeatedly lose track of the walls (lw). See the text for further discussion and Appendix A for a detailed commentary.

In the remainder of this section, we consider the nature of the failures in behavioural integration that occur with levels of λ below or above this intermediate range, and explore the effects of simulated dopamine modulation on the timing and frequency of behaviour switching. Figure 10C provides an analysis of the types of failure of behavioural integration observed at different levels of λ and as described in Table 1. Figure 11A–E shows some example runs, recorded with low and high λ, that help to illustrate the robot behaviour observed at different levels of simulated dopamine.

#### 4.2.2. Behavioural Consequences of Low Simulated Tonic Dopamine (λ < 0.2)

**Slowed movement and periods of inaction**. In Section 3, we showed that the model basal ganglia generates partial (low-efficiency) selection for low levels of simulated dopamine. Since our robotic model employs the basal ganglia output as a gate in targeted motor systems, the consequence of partial selection in behavioural terms should be that this gate is not fully opened for winning competitors; motor acts should be slowed or even extinguished altogether. This expectation, noted in hypothesis 1 above, was borne out in our study (see Figure 10C), which saw the expected translation of partial/weak selection into slowed movement (sm) for all runs at λ level 0.12 or lower. At λ = 0.06 and 0.03 the robot moves too slowly to meet the criterion for successful avoidance (fa) and consequently also fails to complete a successful foraging sequence in the time allowed (ff). Periods during which the robot makes no movement (am), despite being otherwise sufficiently motivated, are seen at λ = 0.06 (an average of 14 s per trial, compared to 2 s for intermediate levels of λ) and for longer spells at λ = 0.03 (an average of 38 s per trial). Note that it is possible to distinguish between the dysfunctional absence of movement due to low λ, as seen in Figure 11A, and its appropriate absence during periods of low motivation (as in the period of no selection for λ = 0.20 in Figure 4). The Supplementary Video (part 4) shows an example of slow movement and no movement for an example run with λ = 0.10.

**Premature deselection**. In the range λ = 0.06–0.15, behaviour can break down as the result of the premature deselection of an ongoing behaviour; this can be seen as a failure of persistence or action maintenance. At λ = 0.09 or below, this typically occurred during the initial wall-seek bout, leading to an absence of movement and failure to reach the wall as noted above. A further point of vulnerability was seen in the range λ = 0.09–0.15 and occurred when the robot attempted to execute the cylinder-pickup FAP but either failed to grasp the cylinder (fgc in Figure 10C) or failed to raise the gripper arm at the end of cylinder-pickup bout (fra in 10C). An example of the fgc failure is shown in the Supplementary Video (part 5). Failure to raise the gripper arm occurred in 80% of trials at λ = 0.12 and 50% of trials at λ = 0.15, and also resulted in a behavioural trap, as described in Appendix A, where the robot detected its lowered arm as an obstacle and engaged in a slow circling behaviour until the end of the trial.

**Failures are more likely at low salience levels**. Our experiments show that, under low λ, weakly selected behaviours are typically not executed with sufficient vigour and can be vulnerable to interruption. Further investigation also shows support for hypothesis 4—that the effects of varying simulated dopamine can also depend on the salience level. Specifically, comparison across the 10 trials at λ = 0.15 shows that the variability in outcome (successful vs. unsuccessful) resulted from differences in the timing of the initial cylinder-pickup bout across trials. Specifically, the robot encountered a cylinder, and initiated the cylinder-pickup FAP, significantly later in the successful runs (M = 66.7 s, SD = 6.88) compared to the unsuccessful runs (M = 52.0 s, SD = 2.23) (independent samples *t*-test: t(4.8) = 4.557, *p* = 0.007; equal variances not assumed). Recall that the salience of cylinder-pickup increases with simulated ‘hunger’, which in turn increases gradually with longer search times. In other words, for those runs at λ = 0.15 in which a cylinder is discovered quickly, and in which the robot is therefore more likely to fail through premature deselection, the selection of the cylinder-pickup behaviour is at a lower salience level than for the successful trials (longer search durations). This can be related to Figure 7B, which shows reduced hysteresis, and hence less behavioural persistence, for low values of λ (compared to intermediate values). More generally, in all low-λ conditions, robot behaviours are executed more efficiently at higher salience levels, and therefore the symptoms of reduced simulated dopamine such as slowed movement are more pronounced when salience is low.

#### 4.2.3. Behavioural Consequences of High Simulated Tonic Dopamine (λ > 0.3)

**Distortion of winning channels by active losers**. At high levels of λ, the non-embodied model predicted reduced inhibition of the motor output from losing channels, leading to distortion of the winning action (hypothesis 3). The behavioural consequences of distortion are visible in the robot model with levels of simulated dopamine of λ ≥ 0.31 and occasionally resulted in behavioural disintegration for λ = 0.31 and 0.34 through failure to complete a foraging bout (ff in Figure 10C). The likelihood of failure increased with very high levels of λ with more than 50% fails at λ = 0.4 and 0.43 and 100% fails at λ = 0.46. At all of these λ levels, failure to forage was typically due to an inability to grasp a cylinder (fgc). However, other evidence of behavioural disintegration was also evident, particularly difficulty in tracking walls (lw). Failure to grasp a cylinder oftens results in a second form of behavioural trap where the robot enters repeated cycles of cylinder-seek and (unsuccessful) cylinder-pickup. An example of this can be seen in Figure 11E (t = 85–120 s), and a further example of this type of failure is shown in the Supplementary Video (part 6).

**Failure is more likely at high salience levels**. That there was a mix of successful and unsuccessful runs, at some high λ levels, indicates that the impact of distortion on behavioural outcome can depend on circumstances. We illustrate this by comparing, in Figure 11D,E, two trials with λ = 0.31, showing that both successful foraging (Figure 11D) and disintegrated foraging (Figure 11E) are possible at this level. In Figure 11D, the robot quickly locates a cylinder at t = 49 s; in Figure 11E, the only unsuccessful run at this λ level, there is a much more protracted cylinder-seek search ending at t = 84 s (see Appendix A for a detailed commentary and comparison). At higher λ levels (0.40 and 0.43), a comparison of successful (M = 37.1 s, SD = 6.06) vs. unsuccessful trials (M = 63.3 s, SD = 16.4) shows that, on average, in successful runs, the robots discovered a cylinder whilst foraging *earlier* than in unsuccessful trials (independent-samples *t*-test: t(18) = −4.741, *p* < 0.001). This is the reverse of the situation with low λ—with high simulated dopamine, there are longer search bouts, giving rise to higher salience levels (from increasing ‘hunger’), that tend to result in greater behavioural disintegration. This again matches hypothesis 3—that the effect of varying simulated dopamine on behaviour will depend upon salience levels, with contrasting effects seen at low and high λ levels.

From Figure 7B, it is evident that we can expect reduced hysteresis (behavioural persistence) for higher levels of λ; however, this figure also shows that increasing salience at high λ does not significantly impact hysteresis. To understand why the robot performs better at lower levels of salience with high λ, we therefore need to look beyond the basal ganglia model itself and to consider the influence of distortion on behavioural persistence via its effect on behaviour. This is the topic of our final analysis.

#### 4.2.4. Effects of Distortion on Behavioural Persistence

A key property of the robotic model, that distinguishes it from the non-embodied simulation, is that selection outcomes have behavioural consequences that shape the robot’s subsequent sensory experiences. More specifically, the robot’s motor output, in part, determines its trajectory through the state space of perceptual and motivational affordances for future selection competitions. Since varying the level of simulated dopamine can influence motor behaviour by slowing movement or by merging partially-selected actions with winning ones, it is interesting to establish whether or not this has any significant consequences for the selection behaviour of the embodied model.

Here, we explore this issue by examining some of the effects of distorted selection on the timing and frequency of behaviour switching. To assist this analysis, an additional 90 robot trials were performed at all of the λ levels previously tested, but this time with a ‘winner-takes-all’ filter applied to the efficiency values of all sub-systems, such that the winning sub-system was always assigned an efficiency of 1.0, and all losers an efficiency of 0.0. In the following analyses, the behaviour of this winner-takes-all variant will be contrasted with the ‘soft’ selection generated by the standard model that allows multiple channels to influence motor output.

**Figure 12:**
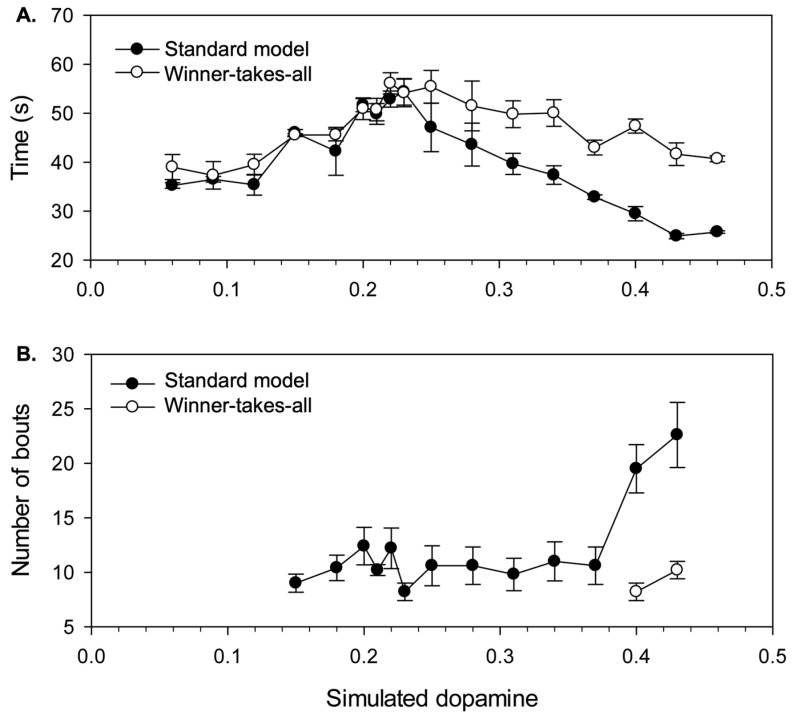
Comparison of the standard ‘soft switching’ robot model of the basal ganglia with a winner-takes-all variant in terms of the timing and frequency of behavioural switching for different levels of simulated dopamine. (**A**) ‘Time-to-switch’ from avoidance to foraging. The plot demonstrates that persistence (time-to-switch to foraging) varies with simulated dopamine and is affected by motor distortion at higher dopamine levels in the case of the standard model only, leading to earlier switching (less persistence) compared with the winner-takes-all variant. (**B**) Total number of bouts during the first avoidance and foraging sequences combined. Bout frequency is significantly increased at very high λ levels for the standard model only, indicating that distortion of motor behaviour can cause more frequent switching. Each average is over five runs. Bars show standard errors.

**Timing of behaviour switching.** Our investigation of the non-embodied model showed significant hysteresis at almost all levels of simulated dopamine in the context of closely matched salience competitions (Figure 7); this should show up strongly in the robot model, in the initial transition from avoidance to foraging behaviour. The key competitors at this point are wall-follow and cylinder-seek and the prime determinant of their relative salience, which eventually allows the latter to prevail, is a gradual, time-determined reduction in ‘fear’ alongside a steady increase in ‘hunger’. The length of time leading up to this switch from avoidance to foraging therefore provides a measure of the operation of behavioural persistence in the model. Figure 12A plots this ‘time-to-switch’ measure against different levels of λ and shows the different outcomes observed with both the standard model (from the original set of 90 trials) and the new winner-takes-all control. For each dopamine level, we plot the average and standard error of the time-to-switch calculated over the five trials.

Comparison with Figure 7B shows that the graph for the winner-takes-all variant provides a good match to the degree of hysteresis found for a fixed salience (on the initial winning channel) of 0.4. Since the salience of wall-follow preceding the switch is typically in the range 0.3–0.4, this demonstrates that hysteresis in the embodied model basal ganglia generates a corresponding level of behavioural persistence under winner-takes-all conditions. However, the standard model generates an interesting difference from this result. Specifically, two-way ANOVA shows a significant interaction (F(1,16) = 3.641, *p* < 0.001) between model type (standard vs. winner-takes-all) and λ. Post hoc comparisons for low, intermediate, and high λ values show a difference for high values only (λ ≥ 0.31) where switching occurs significantly earlier in the standard model (M = 31.7 s, SD = 6.26) compared with that under the winner-takes-all variant (M = 45.4 s, SD = 5.66) (independent samples *t*-test: t(58) = −8.92, *p* < 0.001). We conclude that, with higher λ, the distortion provided by losing channels can significantly reduce behavioural persistence in the robot model. This reduction is over and above that resulting from lower hysteresis in the embedded basal ganglia model.

Looking at Figure 11 (panels D and E), which shows behaviour for two trials with λ = 0.31, we can observe, towards the end of the wall-follow bout (around t = 30), a small, but gradually increasing, output on the cylinder-seek channel. It is this ‘leakage’ of motor output from the cylinder-seek sub-system that constitutes the difference between the standard and winner-takes-all versions of the model. A key to understanding the effect of this distortion is to note that the wall-follow behaviour is not especially robust and is sometimes pushed off-track by sensor noise or wheel slip, even when driven by a clean motor signal. The effect of the motor noise introduced by the partial selection of cylinder-seek is therefore to increase the variability in the robot trajectory, making it more difficult to maintain sensor contact with the nearby wall. In this situation, any loss of the wall percept, due to distorted movement, will lead to a rapid reduction in wall-follow salience and a switch to the cylinder-seek behaviour.

**Increased switching frequency with high simulated dopamine.** If distortion makes some behaviours more vulnerable to interruption, then we might also expect increased levels of behaviour switching. To investigate this possibility, Figure 12B illustrates one specific measure of switch frequency, the total number of bouts occurring during the first avoidance sequence and first foraging sequence of each trial. This measure was preferred to counting bouts (or switches) within a fixed time interval as it allows us to exploit a useful baseline—integrated behaviour (according to our earlier operational definitions) requires a minimum of seven bouts across these two sequences.

Since this measure can only be applied to trials containing a completed foraging sequence, this analysis only considered λ values in the range 0.15–0.43, and the graph plots the average and standard error of the number of bouts observed for the five successful trials at each simulated dopamine level. These data reveal that the performance of the robot is slightly above baseline (seven bouts) across most of the range of simulated dopamine values. However, the number of bouts increases substantially for very high λ levels (λ = 0.40, 0.43; M = 21.3 bouts, SD = 4.73). Moreover, as shown in Figure 12B, when comparing with winner-takes-all selection at these levels (M = 9.2 bouts, SD = 1.99), it is evident that the latter requires significantly fewer bouts (independent samples *t*-test: t(2.22) = 4.33, *p* = 0.041; equal variances not assumed). We therefore conclude that the increased switching seen with the standard model is largely due to the distortion of motor output created by losing competitors. Figure 11E shows an example run with λ = 0.40 that illustrates the increased frequency of bout switching (between wall-seek and wall-follow in t = 0–50 s) that can occur due to distortion with high simulated dopamine.

These analyses of the effects of increased λ on timing and frequency of behavioural switching demonstrate that distortion in the robot model does not inevitably lead to a mixed motor output—attempting to do two things at once. Instead, its effect can be to make certain behavioural states more vulnerable to interruption which can then lead to an increased frequency of behaviour switching.

## 5. Discussion

Robotics can play an important role in neuroscience through its ability to create computational models of the nervous system that are embodied, that is, they control physical devices (robots) that exists in the world. Robotic models are also situated, that is, they must engage in real time and in closed sense–action loops, with the environments in which they are placed [65,66]. Robots, like animals, can display integrated behaviour, where they generate sequences of actions that are coherent with both their internal motivations and the unfolding dynamics of the world [45,67]. Conversely, their behaviour can become disintegrated when action sequences fall out-of-step with the affordances of the environment, and they fail to achieve their goals [36]. The study of robotic models therefore offers opportunities for comparisons with animal and human behaviour that differ from those that are available from the non-embodied models more typically studied in computational neuroscience. For instance, we can study them objectively, as behaving systems, without having to interpret their inputs and outputs [68]. We can also examine the consequences for this observable behaviour of specific interventions that simulate changes to the nervous system studied in relevant animals models, or that might arise in human neurological disorders.

### 5.1. Effects of Simulated Dopamine Modulation on Robot Behaviour

In the current study, we explored the capability of an embedded basal ganglia model to generate patterns of integrated behaviour when operating across a range of simulated tonic dopamine levels (λ). The robot performed the intended avoidance and foraging behaviours successfully for a range of intermediate λ values (0.2–0.28); values below this range caused some slowness of movement, in line with previous predictions from non-embodied models, with movement speeds falling below 75% of its intended vigour at around half of this range (λ = 0.12), and with prolonged periods of no movement for very low λ values (0.06 or less). Some runs with low λ also resulted in the premature deselection of behaviour. High values of λ (0.3 or greater) led to some distortion of motor output as the result of the partial (or full) selection of multiple competing action sub-systems.

We found that simulated dopamine modulation of action selection outside the intermediate range did not invariantly lead to behavioural disintegration, since its effects varied with the precise circumstances of the robot. Specifically, low-λ systems functioned well (selecting cleanly) with high-salience signals but poorly with weak-salience inputs. Conversely, high-λ systems generated cleaner selections at low-salience levels. While expectations from non-embodied modelling (hypotheses 1–4 above) were borne out in the robot implementation, the performance of the robot, across the full range of λ values, was better than might have been predicted from prior analyses of the selection properties of the model basal ganglia. This result can be explained by the finding that the robot, through its behaviour, “self-structures” its own input [69], sampling only a limited area of the state space of salience competitions, and predominantly parts of the space that have better-than-average outcomes (in terms of effective selection).

Hysteresis in the non-embodied model translates into persistence in behavioural expression in the robot. Persistence varied in an interesting way with λ, in a manner only partially explained by the behaviour of the embedded basal ganglia model. Persistence was maximal at intermediate λ levels, with reduced persistence at both lower and higher levels that could be traced to the functioning of the basal ganglia–thalamo-cortical loop. For high λ, reduced persistence was also partly the result of motor distortion, making the current behaviour of the robot more vulnerable to interruption. This is an outcome that was not predictable from the disembodied model. Very high levels of λ also produced an increase in behaviour switching within extended sequences of goal-directed activity. Again, this result is not entirely predicted by the disembodied model, which forecast a greater degree of distortion (mixed behaviour) at high λ values as a result of the partial or full selection of multiple competitors.

### 5.2. The Role of Dopamine in Basal Ganglia Dysfunction in Animals and Humans

Dysfunction of dopaminergic regulation of the basal ganglia is implicated in a range of neurological disorders [35]. In Parkinson’s disease (PD), for instance, tonic dopamine depletion in the striatum is one of the primary drivers of symptoms, including those relating to impaired movement and difficulty in initiating movement [70]. In computational neuroscience models, the progressively debilitating effects of PD have been modelled as attenuation of tonic dopamine in the striatum [71,72]. ADHD, which is characterised by hyperactivity, impulsiveness, impaired attention, and executive dysfunction, has also been linked to dopamine dysregulation, and particularly, to increased levels of dopamine transporter that remove dopamine from the synapse [35]. This outcome has been modelled as resulting in a less pronounced (compared to PD) reduction in striatal dopamine [73]. In schizophrenia, on the other hand, an up-regulation of dopamine is thought to underlie symptoms related to disorganisation, including expression of bizarre or inappropriate behaviour [35,74]. This has been modelled as involving an increase in striatal dopamine [75]. Tourette’s syndrome, which causes sufferers to make involuntary movements or sounds, has also been characterised as a consequence of elevated striatal dopamine [75,76]. Other motor dysfunctions such as chorea and dystonia have been hypothesised to involve a failure to inhibit unwanted movements in which dopamine dysregulation could be implicated [7]. Obsessive compulsive disorder (OCD) is thought to involve hyperactivity in parts of the orbito-frontal cortex, and treatments involving dopamine antagonists have been found to augment the benefits of therapies involving serotonin reuptake inhibitors [77].

A large number of animal models have been developed to investigate the neurological bases for these disorders, many of which have explored genetic, developmental, or drug- or lesion-induced alterations to the dopamine system [77,78,79,80,81,82]. Animal studies have also directly explored the role of dopamine in regulating action selection and motivated behaviour [83,84,85,86]. In the remainder of this discussion, we briefly compare the results of the robot model with findings from animal studies and from studies of human neurological disorders thought to involve lowered or heightened levels of tonic striatal dopamine.

### 5.3. Dopamine-Depleting Interventions and Neurological Conditions Associated with Reduced Striatal Dopamine

**Behaviour execution**. In animals, activational aspects of motivation, such as response rate, vigour, and persistence, are impaired at doses of DA antagonist that leave intact directional or goal-directed aspects of responding (for review see [9,12,16,85]). In patients with PD, major symptoms include slowness in movement (bradykinesia), reduced size of movement (hypokinesia), and absence of movement (akinesia) [87]. Consistent with these findings, in the robot model, slowed movement was a visible consequence as λ was lowered below the intermediate range. This often led to more prolonged bouts of behaviour as action sequences took longer to perform. As λ was further reduced, movements were only partially executed or even fully suppressed, despite high levels of motivation.

**Salience.** In animal models, behaviour evoked by events that have high biological salience are comparatively resistant to dysfunctional dopamine neurotransmission. Thus, complex learned responses to mild stimuli are more prone to disturbance than unlearned responses evoked by intense unconditioned stimuli [12]. Similarly, behaviour directed by external sensory stimuli is less affected than internally motivated behaviour [15,21]. Consummatory behaviours (e.g., eating; drinking) are less disrupted than preparatory behaviours (acts that lead to, or make possible, consummatory behaviours) [10,16,20,88,89]. For example, while lesions of the mesolimbic dopamine projection abolish food hoarding in rats, actual feeding and drinking remain relatively unaffected [89]. High levels of arousal evoked by painful or highly arousing stimuli (such as being plunged into an icy bath) can lead to the restoration of normal behavioural responses (such as swimming) in animals with akinesia caused by lesions that affect the dopamine system [24,90]. Patients with PD often show problems in initiating movement; however, salient visual stimuli such as stripes painted on the floor can facilitate the initiation of walking and reduce the incidence of freezing of gait [91]. Patients with PD can also show “paradoxical kinesia” (close-to-normal movement) in times of acute stress, for example when escaping from fire [92]. Salience competitions appear to have a more marked deleterious effect on patients with PD than on controls. For instance, a stimulus such as a doorway can have an inhibitory effect on movement, causing some patients to freeze. Irrelevant stimuli have also been found to increase reaction times in a manual response task [91]. More broadly, patients with PD can also have difficulty expressing two motor programs simultaneously [87,93].

Our robot model casts interesting light on some of these findings. For instance, we found that, with low λ, behavioural selections made between highly salient competitors were less vulnerable to partial selection, or no selection, than those made on the basis of low-salience competitions (Figure 6). High levels of motivation also led to a general increase in salience for competing behaviours and consequently clean(er) selection. We also found that selection in the low-λ robot was impaired by the increased salience of a competitor, and, in some situations, this led to freezing where competitors were evenly matched (e.g., Supplementary Video, part 4). More generally, at low λ levels, selection of the winning channel was more impacted by the presence of activity in competing channels than under similar circumstances but with λ in the intermediate range.

**Lack of persistence.** Rats with reduced dopamine show difficulty in maintaining motivated behaviour over time. For instance, Gaddy and Neill [17] showed that dopamine-deprived animals had impaired performance of behaviours requiring sustained effort, whilst Salamone [10] found an increased frequency of unfinished feeding bouts (partially eaten food pellets) and failure to carry food pellets to normal feeding loci. Patients with PD often make incomplete movements and can exhibit sudden freezing; they also show rapid fatigue and can have difficulty in maintaining a behaviour over time. For example, in the case of handwriting, for many patients, their letters become smaller and smaller (micrographia) before writing ceases altogether [94]. In the robot model, we found that low λ makes the currently selected behaviour more vulnerable to early deselection or interruption, largely as the result of decreased thalamo-cortical feedback failing to maintain the selected behaviour. A similar challenge could underlie the premature deselection of behaviours seen in PD (see [87]) and the increased distractibility, and lack of persistence, associated with ADHD. As illustrated in Figure 7B, hysteresis in the basal ganglia falls off quite quickly as λ is reduced, including for values in the intermediate range when salience is at a moderate level. This is consistent with the observation that individuals with ADHD show problems with behavioural persistence but without the motor symptoms (bradykinesia, etc.) associated with more profound deficiencies in striatal dopamine.

**Behavioural timing.** Studies with animals provide inconsistent evidence regarding switching frequency and time to initiate behaviours, with outcomes varying with experimental set-up [10]. In the robot model, we found that time to switch depends on the salience of the behaviour and on that of its competitors. This may help explain some inconsistent findings in humans and animals. For example, in PD, there is evidence that while some visual saccades are slowed, others are made more rapidly (hyper-reflexively) than in controls. Through meta-analysis, we previously demonstrated that latency to saccade was dependent on the size (eccentricity) of the saccade, with smaller saccades more likely to be hyper-reflexive [95]. We suggest that this outcome arises because the current fixation behaviour is more vulnerable to early interruption due to reduced hysteresis in the relevant basal ganglia loop.

### 5.4. Dopamine-Increasing Interventions, and Neurological Conditions Involving Increased Striatal Dopamine

**Response frequency and duration.** Animals treated with dopamine agonists show increased response frequencies alongside decreased response durations with increases in dose [96,97,98]. Seen in the context of our robot study, this is consistent with our finding of reduced time to switch and the increase in distractibility and number of bouts with high levels of λ (see Figure 10E and Figure 11).

**Suppressing unwanted actions.** A common feature of neurological disorders involving increased striatal dopamine is difficulty in suppressing unwanted actions and thoughts. These can include the more stereotyped forms of unwanted action or speech seen in Tourette’s syndrome, as well as the short twitch-like movements seen in chorea and thought to resemble fragments of normal behaviours, and perhaps some of the intrusive thoughts and bizarre actions associated with schizophrenia. In the non-embodied basal ganglia model, elevated λ levels resulted in simultaneous selection of multiple channels, an outcome that has some resemblance to dystonia. However, the robot model generated a somewhat different result including patterns of rapid switching between channels, indicating that interruption of ongoing behaviour is made more likely by the motor interference generated by a partially selected competing channel. The more promiscuous forms of selection enabled by higher dopamine levels mean that patterns of behaviour, whose salience activity is “bubbling below the surface”, may find an opportunity for expression due to a momentary loss of attention or concentration.

**Stereotypy and hyperactivity**. At higher doses of DA agonist, animals typically express a narrower range of behaviours and can become fixated on certain action patterns that have become known as stereotypies. These may be oral (e.g., licking, biting, and gnawing) but that can also include forms of repetitive movement, including running [99], that are matched to environmental affordances. For example, Kelley et al. [98], summarising results with a hole board task, commented that “with the higher doses [of amphetamine], locomotor routes become shorter and animals focalize uniquely on the holes (but still maintaining some locomotion and shifting from hole to hole) […] residual components of the original behavior remain, but their pattern is greatly altered” (p. 73). Dopamine transporter (DAT) knockout mice, which have levels of striatal dopamine elevated by 70%, show hyperactivity and reduced habituation when placed in a novel environment [100]. DAT knockout rats, on the other hand, are less sensitive to reward than wildtype animals, and show rigidity of action choice, alongside hyperactivity and compulsive stereotypies [101].

Dopamine agonist-induced stereotypy in animals has been seen as a model for schizophrenia—though schizophrenics typically do not exhibit motor stereotypies, their symptoms often do involve compulsive and repetitive patterns of behaviour and thought [97]. Repetitive sequences of actions, including constrained exploration patterns within an open environment, have been observed in rats treated with the DA agonist quinpirole and have been compared to the rituals seen in people with obsessive compulsive disorder [99].

Qualitatively, the behaviour of the robot model in the highest-λ trials (e.g., Figure 10F) bears some resemblance to patterns of behaviour in hyper-dopaminergic animals—the actions of the robot sample a narrow range of the potential actions and resemble some elements of complete action patterns but are fragmentary, poorly organised, and fail to achieve goals (see, e.g., Supplementary Video, part 6). The underlying cause of the behavioural disintegration is selection (full or partial) of multiple channels, leading to the early interruption of ongoing behaviour or to mixing and distortion of motor acts. In animals, removal of basal ganglia inhibition from the motor system will lead to complex effects as selection of behaviour is governed by multiple brain systems. These include attentional mechanisms, which we might consider as forms of ‘early’ selection, and brainstem and motor mechanisms that may provide forms of ‘late’ selection [102].

### 5.5. Limitations and Related Work

The current model can be improved along a considerable number of lines. First, whilst the Gurney et al. model of basal ganglia employed here has been shown to have enduring appeal (see [103]), there are multiple ways in which it has been improved and extended that could be integrated into a future robot embodiment. For example, a richer model of D1/D2 receptor behaviour (see [104]) could impact the behaviour of a robotic model, as has been investigated for a simulated robot by Bahuguna et al. [105]. Though it is noteworthy that these models, whilst capturing more neurobiological constraints, support the proposition underlying the simpler model deployed here that dopamine, respectively, facilitates/attenuates the cortical input to D1/D2 striatal input neurons. There is also scope to develop the wider architecture. For instance, whilst the current model builds on our understanding of dorsal basal ganglia pathways, the ventral basal ganglia domain shows important similarities and differences, and, significantly, plays a critical role in the regulation of dopamine neurons [106].

Our robotic modelling demonstrates the importance of understanding how selection circuitry interacts with wider sensorimotor systems in the brain. Elsewhere, we have explored this in the context of cortical and sub-cortical loops involved in the selection of eye movements in a robotic active vision model [107], and in the control of whisker-guided behaviour in robots with moving vibrissae [108]. Other interesting work in this direction includes models of basal ganglia interactions with locomotor pattern generator systems such as those underlying fish swimming [109]. For a more complete brain-inspired architecture that includes a basal ganglia model of action selection, see [110].

The current model highlights the importance of understanding how drive systems in the brain interact with action selection mechanisms. In place of the proxy models of drives used here, future models could usefully investigate drive models based on a more realistic model of energy management (e.g., [111]). Another interesting direction to explore is the interaction of the basal ganglia with other brain substrates involved in motivation and action selection. For example, in [112], we developed a layered model of the hypothalamus that models the interplay of hunger and satiety in a simulated foraging task; this model also operates to regulate the activity of simulate dopamine neurons in the ventral tegmental area. Variability in the tonic dopamine signal could be an interesting target for modelling as it is known to be impacted by task engagement, motivation and arousal systems, stress, and reward [113,114,115], and has been shown here to have a significant interaction with salience in supporting effective selection. Finally, action selection is also impacted by other neuromodulators besides dopamine [116], as has been explored in a robotic model by Krichmar [117].

## 6. Conclusions

Neuroscience is faced with the challenge of interpreting the outcomes of animal studies in the context of limited evidence. For instance, in seeking to understand the role of the basal ganglia in action selection, in any given study, whilst we have some access to information about what behaviour is being selected, we generally have very little insight into what competing behaviours are being considered but are not being selected (though see [118] for a study demonstrating such effects on behaviour). Many of our measures of behavioural outcome are also entirely ambiguous with regard to mechanism. For example, perseveration of behaviour (inappropriate repetition) could be as the result of increased salience, increased positive feedback, or the failure of competing behaviours to interrupt. Whilst these alternatives could be disentangled through careful experimentation, the transparency of the robot architecture and the benefits of a synthetic approach (see also [119]) allow us to precisely follow the operation of the underlying control systems and their role in generating observed behaviour [65,68]. Studying robot models can therefore inspire us to think about target brain systems in a new light. For instance, the current robot model reminds us that the activity of non-selected competitors can have a critical influence on how selection competitions are resolved and how the resulting behaviours are expressed.

In our model system, as in animals including humans, we see an inverted U-shape relationship between successful performance of integrated behaviour and the level of tonic (simulated) dopamine. The robot with low simulated dopamine shows slowed movement or no movement, reminiscent of the bradykinesia and akinesia seen in Parkinson’s disease. With excessively high levels of simulated dopamine, the robot displays hyperactivity and rapid switching between behaviours, symptoms that show some resemblance to hyper-dopaminergic outcomes in animals and humans. Perseveration is observed in psychiatric conditions and animal models associated with both reduced and elevated levels of striatal dopamine. Similarly, in our robot model, we saw perseveration with both low and high levels of simulated dopamine, sometimes associated with a behavioural trap. In the latter case, this typically involved the robot failing to complete an ongoing behaviour, leading to repeated cycles of behaviour initiation.

Whilst there is much in this model that is oversimplified, we hope that it demonstrates the potential to apply robotics as a means to test models developed in computational psychiatry. Particularly, the differences between embodied and disembodied simulations investigated here demonstrate that robotics can make observable some of the consequences of computational models that are not apparent when those models are tested in isolation.

## Figures and Tables

**Table 1:** Types of behavioural disintegration in the robot basal ganglia model.

Failure to meet success criterion	
Fails to avoid open space (fa)	Failure with respect to criterion (i) above.
Fails to forage (ff)	Failure with respect to criterion (ii) above.
Behaviours typically leading to fa or ff	
Absence of movement (am)	Failure to express movement despite being motivated. Typically leads to fa as the robot fails to leave open space.
Fails to raise arm (fra)	Fails to lift the arm after grasping a cylinder. Typically leads to ff as the lowered arm blocks the infrared sensor’s ability to detect the environment.
Fails to grasp cylinder (fgc)	Fails to lower the arm sufficiently to grasp a cylinder (therefore grasping at air). This can lead to ff, as, when the robot fails to grasp the cylinder, it then immediately looks for another cylinder. This generally leads to repeated cycles of cylinder-seek followed by (unsuccessful) cylinder-pickup.
Forms of behavioural disintegration typically not leading to fa or ff	
Slowed movement (*sm*)	Scored when behaviour, such as wheeled movement, is slowed to 75% or less of the usual speed (as measured by the output motor signal).
Loses wall (lw)	Losing contact with the wall while expressing wall-follow behaviour. Determined to occur if contact has been lost a minimum of four times in sequence (since occasional losses can occur due to sensor noise).

## Data Availability

The data are available in the Supplementary Materials.

## Supplementary Materials

The following supporting information can be downloaded at https://www.mdpi.com/article/10.3390/biomimetics9030139/s1: Supplementary Methods, Data for Graphs and Tables, and Model Code. The Supplementary Video can be viewed or downloaded from https://zenodo.org/records/10439728 (accessed on 28 December 2023).

## Appendix A. Detailed Commentary on Robot Behaviour in Figure 11

**Low simulated dopamine, Figure 11A–C.**

In Figure 11A, with λ = 0.06 (run 4), the robot starts towards the wall at an extremely slow pace but comes to a standstill a few centimetres short. Some time later, the robot begins (again very slowly) to explore the arena for cylinders. This behavioural pattern can be understood as resulting from the altered competitive relationship between the avoidance and foraging systems in the embedded basal ganglia model. Initially, the salience for wall-seek is high; however, owing to the low level of simulated dopamine, it is only partially selected relative to its competitors. While the robot moves slowly towards the wall, the salience for cylinder-seek (driven by increasing ‘hunger’) begins to increase while that for wall-seek falls away (caused by the programmed reduction in ‘fear’ over time). At the point where the two saliences are close to parity the basal ganglia selection competition is resolved in favour of a stand-off (there is no selection of either action). Movement resumes later when the salience of cylinder-seek has increased further and is sufficient for it to be partially selected.

In Figure 11B, with λ = 0.09 (run 5), the robot completes the avoidance sequence (wall-seek followed by wall-follow), albeit moving slowly; also notice that it is briefly distracted by detecting a cylinder en route (showing that the robot is more distractable than normal). During the subsequent foraging sequence the robot detects the cylinder but the cylinder- pickup bout is affected by slowed movement and the arm is not lowered sufficiently to allow the cylinder to be grasped (*fgc*).

Figure 11C shows an example failure for a run with λ = 0.12 (run 3). The breakdown in behavioural integration occurs at a point (around t = 80), during the execution of the cylinder-pickup bout, where the cylinder has been grasped but the arm has not yet been raised to the vertical position (fra in Figure 10C). Here, the detection of the cylinder in the gripper, combined with the reduced efficiency of the cylinder-pickup selection, brings about a reduction in salience, and loss of positive feedback, which causes that action to be prematurely deselected. After a momentary period of inactivity, and since the robot now holds a cylinder, wall-seek becomes salient and is selected. Unfortunately, the robot now detects its own, still lowered, gripper-arm as a nearby surface and engages in its normal response to this form of sensory input, during a *wall-seek* bout, which is to rotate anti-clockwise (turning out from the ‘wall’). Behaviourally, we observe that the robot engages in a slow anti-clockwise rotation and, since the gripper rotates with the robot and stays down, this leads to a continuous ‘circling’ behaviour. This outcome can be considered to be a form of behavioural trap resulting from circumstances where the robot’s actions serve to maintain sensory inputs that drive a repetitive motor response.

**High simulated dopamine, Figure 11D–F.**

Figure 11D,E show two trials with λ = 0.31 (runs 1 and 5), comparing successful (Figure 11D) and disintegrated (Figure 11E) outcomes.

In the successful run shown in Figure 11D, there are a number of brief episodes of distortion of the selected action (note that the sixth plot from the top shows the distortion level); however, only one of these results in an outcome that is immediately apparent to an observer. This is the distortion of the latter part of a cylinder-pickup bout, through the partial selection of the wall-seek sub-system (this occurs at approximately t = 52 in Figure 11D). The effect of this distortion is, in fact, relatively benign. As described for the low-λ condition discussed above, a close salience competition arises once the cylinder is grasped by the gripper resulting in lowered salience for cylinder-pickup and increased salience for the next element of the foraging sequence, wall-seek. However, whereas this situation results in the reduced efficiency of cylinder-pickup, followed by premature deselection in some low-λ trials; with increased λ, efficiency is not compromised. Instead, cylinder -pickup remains fully selected until the action pattern has completed (raising the arm to the upright position), but wall-seek also begins to control the robot (or more specifically, the wheel motors) before the pickup move is finished. Once again (as in the low-λ trial), the partially raised gripper arm is detected as a nearby surface to which the wall-seek sub-system responds with an anti-clockwise turn. However, since the arm continues to be lifted out of the way by the still-active cylinder-pickup behaviour, there is no behavioural trap. Instead, a smooth transition is observed from the combined turning/lifting movement of the distorted behaviour to the more usual straight-ahead movement generated by wall-seek. In other words, in this instance, significant distortion occurs but does not jeopardise the integrated nature of the full behavioural sequence. Distortion also occurs during the cylinder-deposit behaviour, at around t = 75, this time through the failure to fully deselect the preceding behaviour wall-follow. However, once again, the consequences of the distortion—wheel movements that serve to keep the robot close to the wall—do not interfere with successful completion of the cylinder-deposit bout, and the integrity of the foraging sequence is maintained.

A different outcome occurs in the trial in Figure 11E. Here, after a relatively prolonged bout of search, cylinder-pickup is activated via the detection of a cylinder. However, the selection competition is not cleanly resolved, and the cylinder-seek sub-system is partially selected during repeated bouts of cylinder-pickup. The consequences of this distortion are not benign. Instead, the robot is driven forwards, towards the cylinder, at a point where it needs to move backwards to make room for the lowered gripper arm. As a result, the gripper jaw is not correctly aligned to grasp the cylinder. The usual outcome in this situation is that the cylinder falls from the gripper jaw or is grasped by a thin edge such that its presence is not registered by the optical sensor. In either case, the cylinder-pickup bout is not completed successfully and the robot re-engages the cylinder-seek routine. The appearance of the robot through this episode is of frantic activity—it repeatedly tries to collect a cylinder, but excessive wheel movement means the manoeuvre is never successfully completed. Note that, this time, there *is* a form of behavioural trap—the failure to succeed in the initial cylinder-pickup bout leads to a repeating sequence of alternations between cylinder-seek and cylinder-pickup. Since the goal state of the foraging sequence is never achieved (depositing a cylinder in a ‘nest’ area), the motivation driving these behaviours saturates at a maximum, and the high levels of salience that initiated the distorted output are maintained. Whilst the benign form of distortion (produced by wall-seek) was observed in nearly all trials with dopamine levels of 0.31 ≤ λ ≤ 0.37, the more damaging form (produced by cylinder-seek) was observed in the two fail trials at λ = 0.31, 0.34 and with increased frequency for trials with λ ≥ 0.40.

Figure 11F shows an example run with λ = 0.40 (run 5) that illustrates the increased frequency of bout switching that can occur due to distortion with high simulated dopamine. In this run, the robot has difficulty following the contour of a wall for any extended period, with both the avoidance sequence and the latter part of the foraging sequence including multiple alternating bouts of wall-seek and wall-follow.

## References

[1] Grillner S., Hellgren J., Menard A., Saitoh K., Wikstrom M. (2005). Mechanisms for selection of basic motor programs—Roles for the striatum and pallidum. Trends Neurosci..

[2] Mink J.W. (1996). The basal ganglia: Focused selection and inhibition of competing motor programs. Prog. Neurobiol..

[3] Redgrave P., Prescott T.J., Gurney K. (1999). The basal ganglia: A vertebrate solution to the selection problem?. Neuroscience.

[4] Prescott T.J., Redgrave P., Gurney K. (1999). Layered control architectures in robots and vertebrates. Adapt. Behav..

[5] Balleine B.W., Delgado M.R., Hikosaka O. (2007). The Role of the Dorsal Striatum in Reward and Decision-Making. J. Neurosci..

[6] Grillner S., Robertson B. (2015). The basal ganglia downstream control of brainstem motor centres—An evolutionarily conserved strategy. Curr. Opin. Neurobiol..

[7] Mink J.W. (2003). The basal ganglia and involuntary movements: Impaired inhibition of competing motor patterns. Arch. Neurol..

[8] Schultz W. (2007). Multiple Dopamine Functions at Different Time Courses. Annu. Rev. Neurosci..

[9] Arber S., Costa R.M. (2022). Networking brainstem and basal ganglia circuits for movement. Nat. Rev. Neurosci..

[10] Salamone J.D. (1988). Dopaminergic involvement in activational aspects of motivation—Effects of haloperidol on schedule-induced activity, feeding, and foraging in rats. Psychobiology.

[11] Salamone J.D., Zigmond M.J., Stricker E.M. (1990). Characterization of the impaired feeding-behavior in rats given haloperidol or dopamine-depleting brain-lesions. Neuroscience.

[12] Salamone J.D., Willner P., Scheel-Kruger J. (1991). Behavioral pharmacology of dopamine systems: A new synthesis. The Mesolimbic Dopamine System: From Motivation to Action.

[13] Bakshi V.P., Kelley A.E. (1991). Dopaminergic regulation of feeding-behavior.1. differential-effects of haloperidol microinfusion into 3 striatal subregions. Psychobiology.

[14] Salamone J.D., Mahan K., Rogers S. (1993). Ventrolateral striatal dopamine depletions impair feeding and food handling in rats. Pharmacol. Biochem. Behav..

[15] Bury D., Schmidt W.J. (1987). Effects of systemically and intrastriatally injected haloperidol and apomorphine on grooming, feeding and locomotion in the rat. Behav. Process..

[16] Salamone J.D., Correa M. (2012). The Mysterious Motivational Functions of Mesolimbic Dopamine. Neuron.

[17] Gaddy J.R., Neill D.B. (1977). Differential behavioral changes following intrastriatal application of 6-hydroxydopamine. Brain Res..

[18] Cousins M.S., Salamone J.D. (1996). Involvement of ventrolateral striatal dopamine in movement initiation and execution—A microdialysis and behavioral investigation. Neuroscience.

[19] Cousins M.S., Salamone J.D. (1996). Skilled motor deficits in rats induced by ventrolateral striatal dopamine depletions—Behavioral and pharmacological characterization. Brain Res..

[20] Koob G.F., Riley S.J., Smith S.C., Robbins T.W. (1978). Effects of 6-Hydroxydopamine lesions of the nucleus accumbens septi and olfactory tubercle on feeding, locomotor activity, and amphetamine anorexia in the rat. J. Comp. Physiol. Psychol..

[21] Cools A.R. (1980). Role of the neostriatal dopaminergic activity in sequencing and selecting behavioural strategies: Facilitation of processes involved in selecting the best strategy in a stressful situation. Behav. Brain Res..

[22] Gelissen M., Cools A. (1988). Effect of intracaudate haloperidol and apomorphine on switching motor patterns upon current behavior of cats. Behav. Brain Res..

[23] Marin C., Engber T.M., Bonastre M., Chase T.N., Tolosa E. (1996). Effect of long-term haloperidol treatment on striatal neuropeptides—Relation to stereotyped behavior. Brain Res..

[24] Marshall J.F., Levitan D., Stricker E.M. (1976). Activation-induced restoration of sensorimotor functions in rats with dopamine-depleting brain lesions. J. Comp. Physiol. Psychol..

[25] Teitelbaum P., Schallert T., Whishaw I.Q., Teitelbaum P., Satinoff E. (1983). Sources of spontaneity in motivated behaviour. Handbook of Behavioural Neurobiology.

[26] Oades R.D. (1985). The role of noradrenaline in tuning and dopamine in switching between signals in the cns. Neurosci. Biobehav. Rev..

[27] Iversen S.D., Cools A.R. (1977). Striatal function and stereotyped behaviour. Psychobiology of the Striatum.

[28] Kelley A.E., Iversen S.D. (1979). Substance P infusion into substantia nigra of the rat: Behavioural analysis and involvement of striatal dopamine. Eur. J. Pharmacol..

[29] Bakshi V.P., Kelley A.E. (1991). Dopaminergic regulation of feeding-behavior: 2. differential-effects of amphetamine microinfusion into 3 striatal subregions. Psychobiology.

[30] Langen M., Kas M.J., Staal W.G., van Engeland H., Durston S. (2011). The neurobiology of repetitive behavior: … and men. Neurosci. Biobehav. Rev..

[31] Jankovic J.J., Tolosa E. (2002). Parkinson’s Disease and Movement Disorders.

[32] Moore H., West A.R., Grace A.A. (1999). The regulation of forebrain dopamine transmission: Relevance to the pathophysiology and psychopathology of schizophrenia. Biol. Psychiatry.

[33] Brisch R., Saniotis A., Wolf R., Bielau H., Bernstein H.G., Steiner J., Bogerts B., Braun K., Jankowski Z., Kumaratilake J. (2014). The Role of Dopamine in Schizophrenia from a Neurobiological and Evolutionary Perspective: Old Fashioned, but Still in Vogue. Front. Psychiatry.

[34] Joel D. (2006). Current animal models of obsessive compulsive disorder: A critical review. Prog. Neuro-Psychopharmacol. Biol. Psychiatry.

[35] Klein M.O., Battagello D.S., Cardoso A.R., Hauser D.N., Bittencourt J.C., Correa R.G. (2019). Dopamine: Functions, Signaling, and Association with Neurological Diseases. Cell. Mol. Neurobiol..

[36] Prescott T.J., González F.M.M., Gurney K., Humphries M.D., Redgrave P. (2006). A robot model of the basal ganglia: Behaviour and intrinsic processing. Neural Netw..

[37] Montague P.R., Dolan R.J., Friston K.J., Dayan P. (2012). Computational psychiatry. Trends Cogn. Sci..

[38] Tolu S., Strohmer B., Zahra O. (2023). Perspective on investigation of neurodegenerative diseases with neurorobotics approaches. Neuromorphic Comput. Eng..

[39] Gurney K., Prescott T.J., Redgrave P. (2001). A computational model of action selection in the basal ganglia: I. A new functional anatomy. Biol. Cybern..

[40] Gurney K., Prescott T.J., Redgrave P. (2001). A computational model of action selection in the basal ganglia: II. Analysis and simulation of behaviour. Biol. Cybern..

[41] Humphries M.D., Gurney K. (2002). The role of intra-thalamic and thalamocortical circuits in action selection. Netw. Comput. Neural Syst..

[42] McFarland D., Bosser T. (1993). Intelligent Behaviour in Animals and Robots.

[43] Snaith S., Holland O., Meyer J.-A., Wilson S. (1990). An investigation of two mediation strategies suitable for behavioural control in animals and animats. From Animals to Animats: Proceedings of the First International Conference Simulation of Adaptive Behaviour.

[44] Prescott T.J. (2008). Action Selection. Scholarpedia.

[45] Prescott T.J. (2007). Forced moves or good tricks in design space? Landmarks in the evolution of neural mechanisms for action selection. Adapt. Behav..

[46] Ludlow A.R. (1983). Applications of Computer Modelling to Behavioural Coordination. Ph.D. Thesis.

[47] McFarland D. (1989). Problems of Animal Behaviour.

[48] McFarland D.J., Sibly R.M. (1975). The behavioural final common path. Philos. Trans. R. Soc. B.

[49] Gillies A., Willshaw D. (2004). Models of the subthalamic nucleus: The importance of intranuclear connectivity. Med. Eng. Phys..

[50] Parent A., Hazrati L.N. (1995). Functional anatomy of the basal ganglia. II. The place of subthalamic nucleus and external pallidum in basal ganglia circuitry. Brain Res. Brain Res. Rev..

[51] Suryanarayana S.M., Kotaleski J.H., Grillner S., Gurney K.N. (2019). Roles for globus pallidus externa revealed in a computational model of action selection in the basal ganglia. Neural Netw..

[52] Gerfen C.R., Surmeier D.J. (2011). Modulation of Striatal Projection Systems by Dopamine. Annu. Rev. Neurosci..

[53] Akkal D., Burbaud P., Audin J., Bioulac B. (1996). Responses of substantia nigra pars reticulata neurons to intrastriatal D1 and D2 dopaminergic agonist injections in the rat. Neurosci. Lett..

[54] Cui G., Jun S.B., Jin X., Pham M.D., Vogel S.S., Lovinger D.M., Costa R.M. (2013). Concurrent activation of striatal direct and indirect pathways during action initiation. Nature.

[55] Tecuapetla F., Jin X., Lima S.Q., Costa R.M. (2016). Complementary Contributions of Striatal Projection Pathways to Action Initiation and Execution. Cell.

[56] González F.M., Prescott T.J., Gurney K., Humphries M., Redgrave P., Meyer J.A. (2000). An embodied model of action selection mechanisms in the vertebrate brain. From Animals to Animats 6: Proceedings of the 6th International Conference on the Simulation of Adaptive Behavior.

[57] Hinde R.A. (1966). Animal Behaviour: A Synthesis of Ethology and Comparative Psychology.

[58] Lorenz K. (1935). Der Kumpan in der Umwelt des Vogels. J. Ornithol..

[59] Schultz W., Dayan P., Montague P.R. (1997). A neural substrate for prediction and reward. Science.

[60] Blomfield S. (1974). Arithmetical operations performed by nerve cells. Brain Res..

[61] Koch C., Poggio T., Torre V. (1983). Nonlinear interactions in a dendritic tree: Localization, timing, and role in information processing. Proc. Natl. Acad. Sci. USA.

[62] Tsumori T., Yasui Y. (1997). Organization of the nigro-tecto-bulbar pathway to the parvicellular reticular formation: A light- and electron-microscopic study in the rat. Exp. Brain Res..

[63] A Rossi M., E Li H., Lu D., Kim I.H., A Bartholomew R., Gaidis E., Barter J.W., Kim N., Cai M.T., Soderling S.H. (2016). A GABAergic nigrotectal pathway for coordination of drinking behavior. Nat. Neurosci..

[64] Lehner P.N. (1996). Handbook of Ethological Methods.

[65] Prescott T.J., Wilson S.P. (2023). Understanding brain functional architecture through robotics. Sci. Robot..

[66] Hallam J.C., Malcolm C.A. (1994). Behaviour: Perception, action and intelligence—The view from situated robotics. Philos. Trans. R. Soc. London. Ser. A Phys. Eng. Sci..

[67] Brooks R.A., Cliff D., Husbands P., Meyer J.-A., Wilson S.W. (1994). Coherent behaviour from many adaptive processes. From Animals to Animats 3: Proceedings of the Third International Conference on the Simulation of Adaptive Behaviour.

[68] Prescott T.J., Ayers J.O.S.E.P.H., Grasso F.W., Verschure P.F., Arbib M.A., Bonaiuto J.J. (2016). Embodied models and neurorobotics. From Neuron to Cognition via Computational Neuroscience.

[69] Verschure P.F.M.J., Voegtlin T., Douglas R.J. (2003). Environmentally mediated synergy between perception and behaviour in mobile robots. Nature.

[70] Obeso J.A., Marin C., Rodriguez-Oroz C., Blesa J., Benitez-Temiño B., Mena-Segovia J., Rodríguez M., Olanow C.W. (2008). The basal ganglia in Parkinson’s disease: Current concepts and unexplained observations. Ann. Neurol..

[71] Frank M.J. (2005). Dynamic dopamine modulation in the basal ganglia: A neurocomputational account of cognitive deficits in medicated and nonmedicated Parkinsonism. J. Cogn. Neurosci..

[72] Guthrie M., Myers C.E., Gluck M.A. (2009). A neurocomputational model of tonic and phasic dopamine in action selection: A comparison with cognitive deficits in Parkinson’s disease. Behav. Brain Res..

[73] Frank M.J., Santamaria A., O’Reilly R.C., Willcutt E. (2007). Testing Computational Models of Dopamine and Noradrenaline Dysfunction in Attention Deficit/Hyperactivity Disorder. Neuropsychopharmacology.

[74] Sonnenschein S.F., Gomes F.V., Grace A.A. (2020). Dysregulation of Midbrain Dopamine System and the Pathophysiology of Schizophrenia. Front Psychiatry.

[75] Maia T.V., Conceicao V.A. (2017). The Roles of Phasic and Tonic Dopamine in Tic Learning and Expression. Biol. Psychiatry.

[76] Singer H.S., Szymanski S., Giuliano J., Yokoi F., Dogan A.S., Brasic J.R., Zhou Y., Grace A.A., Wong D.F. (2002). Elevated Intrasynaptic Dopamine Release in Tourette’s Syndrome Measured by PET. Am. J. Psychiatry.

[77] Xue J., Qian D., Zhang B., Yang J., Li W., Bao Y., Qiu S., Fu Y., Wang S., Yuan T.-F. (2022). Midbrain dopamine neurons arbiter OCD-like behavior. Proc. Natl. Acad. Sci. USA.

[78] Jones C.A., Watson D.J.G., Fone K.C.F. (2011). Animal models of schizophrenia. Br. J. Pharmacol..

[79] Betarbet R., Sherer T.B., Greenamyre J.T. (2002). Animal models of Parkinson’s disease. BioEssays.

[80] Blesa J., Przedborski S. (2014). Parkinson’s disease: Animal models and dopaminergic cell vulnerability. Front. Neuroanat..

[81] Dawson T.M., Ko H.S., Dawson V.L. (2010). Genetic Animal Models of Parkinson’s Disease. Neuron.

[82] Schober A. (2004). Classic toxin-induced animal models of Parkinson’s disease: 6-OHDA and MPTP. Cell Tissue Res..

[83] Wise R.A. (2004). Dopamine, learning and motivation. Nat. Rev. Neurosci..

[84] Ikemoto S., Panksepp J. (1999). The role of nucleus accumbens dopamine in motivated behavior: A unifying interpretation with special reference to reward-seeking. Brain Res. Rev..

[85] Salamone J.D., Correa M., Yang J.-H., Rotolo R., Presby R. (2018). Dopamine, Effort-Based Choice, and Behavioral Economics: Basic and Translational Research. Front. Behav. Neurosci..

[86] Berridge K.C. (2012). From prediction error to incentive salience: Mesolimbic computation of reward motivation. Eur. J. Neurosci..

[87] Berardelli A., Rothwell J.C., Thompson P.D., Hallett M. (2001). Pathophysiology of bradykinesia in Parkinson’s disease. Brain.

[88] Blackburn J.R., Phillips A.G., Fibiger H.C. (1987). Dopamine and preparatory behavior: 1. effects of pimozide. Behav. Neurosci..

[89] Kelley A.E., Stinus L. (1985). Dissapearance of hoarding behavior after 6-hydroxydopamine lesions of the mesolimbic dopamine neurons and its reinstatement with l-dopa. Behav. Neurosci..

[90] Keefe K.A., Salamone J.D., Zigmond M.J., Stricker E.M. (1989). Paradoxical kinesia in parkinsonism is not caused by dopamine release. Studies in an animal model. Arch. Neurol..

[91] McDowell S.-A., Harris J. (1997). Irrelevant peripheral visual stimuli impair manual reaction times in Parkinson’s disease. Vis. Res..

[92] Schwab R.S. (1972). Akinesia paradoxica. Electroencephalogr. Clin. Neurophysiol..

[93] Benecke R., Rothwell J.C., Dick J.P.R., Day B.L., Marsden C.D. (1986). Performance of simultaneous movements in patients with parkinson’s disease. Brain.

[94] Shukla A.W., Ounpraseuth S., Okun M.S., Gray V., Schwankhaus J., Metzer W.S. (2012). Micrographia and related deficits in Parkinson’s disease: A cross-sectional study. BMJ Open.

[95] Chambers J.M., Prescott T.J. (2010). Response times for visually guided saccades in persons with Parkinson’s disease: A meta-analytic review. Neuropsychologia.

[96] Rebec G.V., Bashore T.R. (1984). Critical issues in assessing the behavioral effects of amphetamine. Neurosci. Biobehav. Rev..

[97] Seiden L.S., Sabol K.E., Ricaurte G.A. (1993). Amphetamine: Effects on Catecholamine Systems and Behavior. Annu. Rev. Pharmacol. Toxicol..

[98] Kelley A.E., Winnock M., Stinus L. (1986). Amphetamine, apomorphine and investigatory behavior in the rat: Analysis of the structure and pattern of responses. Psychopharmacology.

[99] Eilam D. (2017). From an animal model to human patients: An example of a translational study on obsessive compulsive disorder (OCD). Neurosci. Biobehav. Rev..

[100] Zhuang X., Oosting R.S., Jones S.R., Gainetdinov R.R., Miller G.W., Caron M.G., Hen R. (2001). Hyperactivity and impaired response habituation in hyperdopaminergic mice. Proc. Natl. Acad. Sci. USA.

[101] Cinque S., Zoratto F., Poleggi A., Leo D., Cerniglia L., Cimino S., Tambelli R., Alleva E., Gainetdinov R.R., Laviola G. (2018). Behavioral Phenotyping of Dopamine Transporter Knockout Rats: Compulsive Traits, Motor Stereotypies, and Anhedonia. Front Psychiatry.

[102] Allport A., Heuer H., Sanders A.F. (1987). Selection for action: Some behavioial and neurophysiological considerations of attention and action. Perspectives on Perception and Action.

[103] Humphries M.D., Gurney K. (2021). Making decisions in the dark basement of the brain: A look back at the GPR model of action selection and the basal ganglia. Biol. Cybern..

[104] Humphries M.D., Lepora N., Wood R., Gurney K. (2009). Capturing dopaminergic modulation and bimodal membrane behaviour of striatal medium spiny neurons in accurate, reduced models. Front. Comput. Neurosci..

[105] Bahuguna J., Weidel P., Morrison A. (2019). Exploring the role of striatal D1 and D2 medium spiny neurons in action selection using a virtual robotic framework. Eur. J. Neurosci..

[106] Humphries M.D., Prescott T.J. (2010). The ventral basal ganglia, a selection mechanism at the crossroads of space, strategy, and reward. Prog. Neurobiol..

[107] Cope A.J., Chambers J.M., Prescott T.J., Gurney K.N. (2017). Basal Ganglia Control of Reflexive Saccades: A Computational Model Integrating Physiology Anatomy and Behaviour. bioRxiv.

[108] Prescott T.J., Mitchinson B., Lepora N.F., Wilson S.P., Anderson S.R., Porrill J., Dean P., Fox C.W., Pearson M.J., Sullivan J.C., Krieger P., Groh A. (2015). The robot vibrissal system: Understanding mammalian sensorimotor co-ordination through biomimetics. Sensorimotor Integration in the Whisker System.

[109] Sarvestani I.K., Kozlov A., Harischandra N., Grillner S., Ekeberg Ö. (2013). A computational model of visually guided locomotion in lamprey. Biol. Cybern..

[110] Verschure P.F.M.J., Pennartz C.M.A., Pezzulo G. (2014). The why, what, where, when and how of goal-directed choice: Neuronal and computational principles. Philos. Trans. R. Soc. Lond. B Biol. Sci..

[111] Girard B., Tabareau N., Pham Q., Berthoz A., Slotine J.-J. (2008). Where neuroscience and dynamic system theory meet autonomous robotics: A contracting basal ganglia model for action selection. Neural Netw..

[112] Jimenez-Rodriguez A., Prescott T.J. (2023). Motivational Modulation of Consummatory Behaviour and Learning in a Robot Model of Spatial Navigation. Biomimetic and Biohybrid Systems.

[113] Marinelli M., McCutcheon J.E. (2014). Heterogeneity of dopamine neuron activity across traits and states. Neuroscience.

[114] Rice M.E., Patel J.C., Cragg S.J. (2011). Dopamine release in the basal ganglia. Neuroscience.

[115] Goto Y., Otani S., Grace A.A. (2007). The Yin and Yang of dopamine release: A new perspective. Neuropharmacology.

[116] Krichmar J.L. (2008). The neuromodulatory system: A framework for survival and adaptive behavior in a challenging world. Adapt. Behav..

[117] Krichmar J. (2013). A neurorobotic platform to test the influence of neuromodulatory signaling on anxious and curious behavior. Front. Neurorobotics.

[118] Wang Z., Jusup M., Shi L., Lee J.-H., Iwasa Y., Boccaletti S. (2018). Exploiting a cognitive bias promotes cooperation in social dilemma experiments. Nat. Commun..

[119] Hommel B., Chapman C.S., Cisek P., Neyedli H.F., Song J.-H., Welsh T.N. (2019). No one knows what attention is. Atten. Percept. Psychophys..

