# Supplementary material for: Simulated Dopamine Modulation of a Neurorobotic Model of the Basal Ganglia
Source: Biomimetics (Basel). 2024 Feb 25;9(3):139. doi: 10.3390/biomimetics9030139 (PMC10967936; doi:10.3390/biomimetics9030139)
Supplement: Supplementary file 1 [file biomimetics-09-00139-s001.zip › Prescott_et_al_supplementary_materials/Model Code/README.pdf]

Simulated dopamine modulation of a neurorobotic model of the basal ganglia. MDPI Biomimetics, 2024.
Authors: Tony J. Prescott, Fernando M. Montes Gonzalez, Kevin Gurney, Mark D. Humphries, and Peter Redgrave.

SOURCE CODE

1. All code is in written C++, and authored by Tony J. Prescott and Fernando Montes.

2. This code is made available on a CC_BY license that allows free use and modification. Please cite the authors. No warranty is offered or implied. Most files include some comments, for an overview of the system see Supplementary_Methods. This code is provided FOR INFORMATION AND RESEARCH USE ONLY we are unable to provide support for third-party use of this software.

3. The basal ganglia model can be run in standalone model using the “bg” and “test_bg” libraries. The header file “bg/bg_extended_f.h” includes the version of the extended basal ganglia discussed/tested in the article.

4. All other files and folders relate to the embedded model. This code requires Webots 2.0 from www.cyberbotics.com to run in simulation, the WEBOTS_HOME library to be installed (see “makefile”) including relevant header files for Khepera 1 robot (see “robot/motor_plant.h”). The GUI was developed for the Unix operating system. The main control loop is in “biras.cpp” (BIRAS= Brain Inspired Robot Action Selection).

5. For the physical robot, the Khepera gripper module is required. The code is designed to operate the robot via an umbilical cable and has not been tested for remote operation.

Contents of Folders

FOLDER: BG
afferent.cpp: methods for the afferent class (defined in basal_ganglia.h)
basal_ganglia.h: basal ganglia class and methods
bg/bg_basic_f.h: Method for customising the basic basal ganglia model
bg/bg_extended.h: Method for customising the extended basal ganglia model
da_leaky.h: Dopamine (DA) modulation for leaky integrator neurons
leaky.h: Leaky neuron class
nucleus.cpp: Nucleus class and methods

FOLDER: CTRL
controller_gui.h: gui_flag and controller_gui classes
controller.h: class for controlling experiments
ctrl/ctrl_motivated.h: Controller method for customising model (sets parameters for robot and embedding architecture)
experiment.h: Controller methods for running/analysing a robot experiment

ctrl/wall_follower.h: Controller method for simpler wall-following robot

FOLDER: ROBOT
behaviour.h: class definition for action subsystems
forage.h: action sub-system classes for Khepera 'foraging' behaviour
motivation.h: motivational sub-systems
motor_plant.h: motor post-processing functions
percept.h: perceptual sub-systems
robot.h: definitions for robot interface
sensory_sys.h: sensory sub-systems
subsys.h: base class of basal ganglia input subsystems

FOLDER: TEST_BG
benchmark.cpp: test/benchmark program
grad.cpp: test/benchmark program for gradually changing salience inputs
mult.cpp: test/benchmark program for multi-channel (5) salience inputs
step.cpp: test/benchmark program for step change salience inputs
selection.cpp: test/benchmark program for gradual change with hysteresis

FOLDER: UI
ethogram_window.h: window class for displaying behavioural activity
graph_window.h: window class for displaying graphs
interface.h: class for managing all windows
iwindow.h: base window class for user interface
monitor_window.h: window class for showing bg nuclei activation
observer_log.h: class and methods for generating Observer Pro data files
percepts_window.h: window class for displaying perceptual variables
salience_window.h: window class for displaying behavioural salience
percepts_window.h: window class for displaying perceptual variables
percepts_window.h: window class for displaying perceptual variables
tools_window.h: window class for online control of model basal ganglia

FOLDER: UTILS
bout.h: object for storing record of behavioural data
bv_stat.h: stores contour plot data for a bivariate variable
data_log.h: class and methods for logging model activity
io.h: stores and calculates statistics relating to a variable
timer.h: timer and timer_stat classes
