# Supplementary material for: Simulated Dopamine Modulation of a Neurorobotic Model of the Basal Ganglia
Source: Biomimetics (Basel). 2024 Feb 25;9(3):139. doi: 10.3390/biomimetics9030139 (PMC10967936; doi:10.3390/biomimetics9030139)
Supplement: Supplementary file 1 [file biomimetics-09-00139-s001.zip › Prescott_et_al_supplementary_materials/Supplementary Methods.pdf]

Simulated dopamine modulation of a neurorobotic model of the basal ganglia

Tony J. Prescott, Fernando M. Montes Gonzalez, Kevin Gurney, Mark D. Humphries, and Peter Redgrave.
Biomimetics, 2024.

Supplementary Methods: Implementation of the Robot Basal Ganglia Model

The difference components of the robot basal ganglia model are described in the following sections as labelled in figure S1. See Prescott et al. (2006) (ref 36), Gurney et al. (2000) (ref. 40), and Humphries and Gurney (2002) (ref. 41) for further information. Full code (in C++) is provided in the Supplementary Materials.

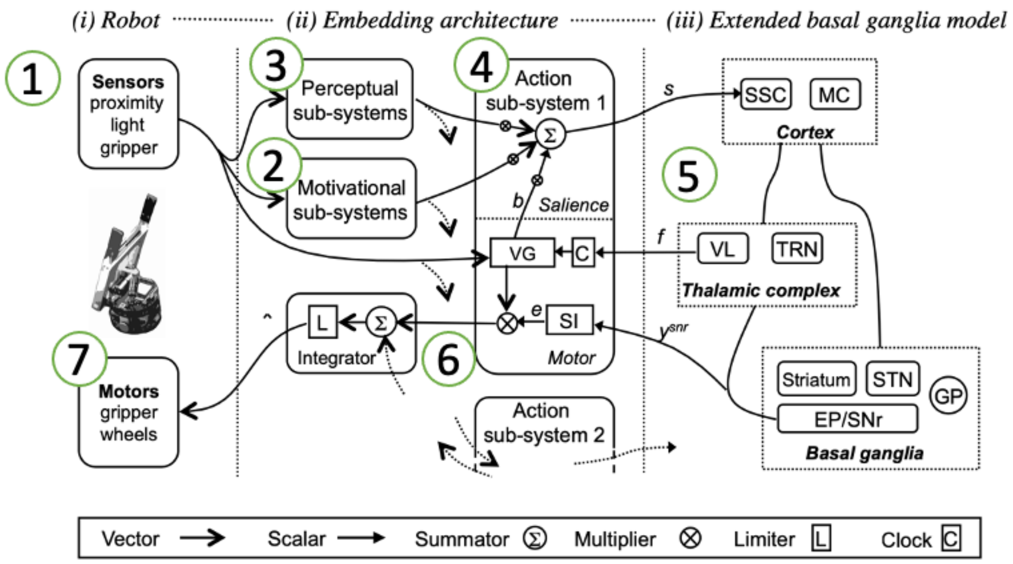

Figure S1. The embedded robot basal ganglia model. The robot (i) interfaces via the embedding architecture (ii) with the extended basal ganglia model (iii). Reproduced with permission from Prescott et al. (ref. 36). 2006, Elsevier Science and Engineering Journals.

1. Sensors

The *Khepera* robot has 8 peripheral sensors. The first six (1–6) are arranged in a semi-circle at the front of the robot, sensor 1 is furthest left, sensor 6 furthest right, with sensors 3 and 4 covering a narrow field of view directly ahead of the robot. Sensors 7 and 8 are directed towards the rear of the robot and are not used in the current model. The *i*th peripheral sensor generates both an infra-red proximity reading, *ir(i)*, which is integer valued in the range 0-1023 with higher values indicating greater proximity to a nearby surface, and an ambient-light reading, *amb(i)*, in the range 0–450 with lower values indicate greater luminance. The optical gripper sensor, *opt()*, provides a binary signal, 1 if there is an object in the gripper, 0 otherwise. The arm position sensor, *arm(),* returns a value in the range 255 (lowered in front) to 152 (raised overhead). We calculate the following variables using the current infrared and ambient light readings for use in calculating perceptual and motivational variables and determining motor vector values as described further below.

$$ir_{tot} = \sum_{i=1}^{6} ir(i),\ \ ir_{left} = \sum_{i=1}^{3} ir(i),\ \ ir_{right} = \sum_{i=4}^{6} ir(i),$$

$$ir_{diff} = \left|ir_{left} - ir_{right}\right|, side = \begin{cases} \text{left: } ir_{left} \geq ir_{right} \\ \text{right: otherwise} \end{cases},$$

$$detect(i) = \begin{cases} 1: ir(i) > 30 \\ 0: \text{otherwise} \end{cases}, n_{touch} = \sum_{i=1}^{6} detect(i),$$

$$lit(i) = \begin{cases} 1: amb(i) < 100 \\ 0: \text{otherwise} \end{cases}, n_{lit} = \sum_{i=1}^{6} lit(i).$$

2. Motivational Sub-systems

There are two motivational sub-systems, m_{fear} and m_{hung} modelling fear and hunger respectively. m_{fear} is initialised to 1.0 and decays at a rate of -0.0007/time-step towards a minimum value of 0.0. m_{hung} is initialised to 0.2 and increases at a rate of 0.0015/time-step towards a maximum value of 1.0. On any time-step in which a cylinder is deposited in a 'nest' area (an illuminated corner of the arena) m_{hung} is immediately set to 0.0.

3. Perceptual Sub-systems

The four perceptual systems in the embedding architecture detect the presence of a wall (p_{wall}), a nest (p_{nest}), a cylinder (p_{cyl}), and the status of the gripper (p_{grip}). Each delivers a value of either +1 or -1 at each time-step.

Wall-detection. A wall detected (p_{wall}= +1) only when the following conditions are met: (i) the sum of infrared readings across all forward-facing sensors, ir_{tot}, is greater than 800 indicating a nearby surface, and either (ii) the left-most or right-most sensors indicates close proximity to a surface on that side (ir(1)>800 or ir(6)>800), or (iii) three or more of the forward facing sensors detect a surface at any distance (n_{touch}>3). The wall percept also requires that the arm is raised above the horizontal level (arm() <=227) since it may otherwise be detected as a surface by the infrared sensors. Note that action sub-systems may still detect the arm as a surface when it is lowered when calculating motor commands. These conditions can be summarised as:

$$p_{wall} = \begin{cases} +1\text{: } (ir_{tot} > 800) \wedge \left(r(1) > 800 \ \vee \ ir(6) > 800 \ \vee \ \sum_{i=1}^{6} detect(i) \geq 3 \right) \wedge (arm() \leq 227) \\ -1\text{: otherwise} \end{cases}.$$

Detecting a nest. A nest area (an illuminated corner of the arena) is detected if the ambient light reading on two or more forward peripheral sensors is below threshold, i.e.

$$p_{nest} = \begin{cases} +1\text{: } n_{lit} \geq 2 \\ -1\text{: otherwise} \end{cases}.$$

Detecting a cylinder. The presence of a cylinder is indicated when the front-most sensors, 3 and 4, detect a surface at close proximity, and the two sensors on either side (2 and 5) do not detect a surface. A cylinder cannot be detected if the nest percept is true. Hence,

$$p_{cyl} = \begin{cases} +1\text{: } (ir(2) < 10) \wedge (ir(3) > 1000) \wedge (ir(4) > 1000) \wedge (ir(5) < 10) \wedge (p_{nest} \neq 1) \\ -1\text{: otherwise} \end{cases}$$

Gripper status. If the optical sensor in the gripper is triggered then the gripper is considered to be holding an object, hence

$$p_{grip} = \begin{cases} +1\text{: } grip() = 1 \\ -1\text{: otherwise} \end{cases}.$$

4. Action Sub-system Saliency

The salience s_i of the ith sub-system is calculated as a weighted sum of relevant perceptual and motivational variables and a threshold term. The model also allows an action sub-system to contribute to its own salience by generating a signal, termed the *busy signal*, indicating the urgency or importance attached to completing an ongoing task (indicated by the symbol b in figure S1). Details of busy signal calculation for specific action sub-systems are provided in table S1. The weights in the following equations were selected by hand and fine-tuned by observing the behaviour of the robot.

Cylinder-seek: $s_1 = s_{seek} = -0.12p_{cyl} - 0.12p_{grip} - 0.06m_{fear} + 0.45m_{hung}$.

Cylinder-pickup: $s_2 = s_{pick} = 0.21p_{cyl} - 0.15p_{grip} - 0.18m_{fear} + 0.18m_{hung} + 0.78b_{pick} + 0.25$.

Wall-seek: $s_3 = s_{wall} = -0.12p_{wall} + 0.14p_{grip} + 0.18m_{fear} + 0.25$.

Wall-follow: $s_4 = s_{foll} = 0.12p_{wall} + 0.14p_{grip} + 0.21m_{fear} + 0.25b_{foll} + 0.25.$

Cylinder-deposit: $s_5 = s_{dep} = 0.33p_{nest} + 0.33p_{grip} + 0.18m_{hung} + 0.40b_{dep} + 0.13.$

5. The Extended Basal Ganglia Model

In each robot time-step, the embedded basal ganglia is initialised with the current salience values of the action sub-systems and then run to convergence as described below. Within the embedded model we use a standard leaky integrator unit defined as follows. Let *a* be the activation of a component of the model and *u* its net input. We assume a rate constant, k, that corresponds to cell membrane capacitance/resistance. The change in activation $\dot{a} = da/dt$ is then given by:

$$\dot{a} = -k(a - u). \quad (1)$$

The output of the unit, y, which corresponds to mean firing rate, is constrained to lie between 0 and 1 and is given by the piecewise linear function:

$$y = L(a, \theta) = \begin{cases} 0: a < \theta \\ (a - \theta): \theta \leq a \leq 1/(1+\theta). \\ 1: a > (1 + \theta) \end{cases} \quad (2)$$

Here θ is a threshold such that any value of *a* which is less than θ generates an output of zero. A negative value for θ therefore indicates tonic activation and a positive value indicates resistance to synaptic input. The following equations define the net input, *u,* and output, *y,* for the *i*th channel in each component nucleus of the extended model.

Somatosensory cortex (*ssc*): $u_i^{ssc} = s_i, y_i^{ssc} = L(a_i^{ssc}, 0.0).$

Motor cortex (*mc*): $u_i^{mc} = y_i^{ssc} + y_i^{vl},\ y_i^{mc} = L(a_i^{mc}, 0.0).$

Striatum D1 (*d1*): $u_i^{d1} = (1 + \lambda)\frac{1}{2}(y_i^{ssc} + y_i^{mc}),\ y_i^{d1} = L\left(a_i^{d1}, 0.2\right).$

Striatum D2 (*d2*): $u_i^{d2} = (1 - \lambda)\frac{1}{2}(y_i^{ssc} + y_i^{mc}),\ y_i^{d2} = L\left(a_i^{d2}, 0.2\right).$

Subthalamic nucleus (*stn*): $u_i^{stn} = \frac{1}{2}(y_i^{ssc} + y_i^{mc}) - y_i^{gp},\ \ y_i^{stn} = L(a_i^{stn}, -0.25).$

Globus pallidus (*gp*): $u_i^{gp} = 0.9\sum_i y_i^{stn} - y_i^{d2},\ y_i^{gp} = L(a_i^{gp}, -0.2).$

Substantia nigra pars reticulata (*snr*): $u_i^{snr} = 0.9\sum_i y_i^{stn} - y_i^{d1} - 0.3y_i^{gp},\ y_i^{snr} = L(a_i^{snr}, -0.2).$

Ventrolateral thalamus (*vl*): $u_i^{vl} = y_i^{mc} - y_i^{snr} - \left(0.125y_i^{trn} + 0.4\sum_{j\neq i} y_j^{trn}\right), y_i^{vl} = L(a_i^{vl}, 0.0).$

Thalamic reticular nucleus (*trn*): $u_i^{trn} = y_i^{mc} + y_i^{vl} - 0.2y_i^{snr},\ y_i^{trn} = L(a_i^{trn}, 0.0).$

The time course of the model was simulated with a Euler update rule to provide a discrete time approximation to equation 1:

$$a_i(t) = a_i(t-1) + \Delta a_i(t), \text{ where} \quad (3)$$

$$\Delta a_i(t) = -k(a_i(t-1) - u_i(t))\Delta t.$$

In this approximation, the net input, $u_i(t)$ to each channel is calculated, as indicated in the equations above. These use the outputs $y_i(t-1)$ of the relevant components from the previous iteration and the salience value, s_i, provided by the relevant action sub-system (which is fixed throughout convergence). The activation, $a_i(t)$, is then updated using equation 3, and output, $y_i(t)$, calculated by substituting $a_i(t)$ in equation 2 with the appropriate threshold. A step-size of $\Delta t = 0.015$ and rate constant $k = 20$ were used in the experiments reported here. Convergence was defined as two consecutive time-steps in which the smallest Δa was less than 0.0001. The model typically converged in between 2 and 300 time-steps.

6. Action Output, Gating and Integration

The Khepera robot has two driven wheels, a gripper arm that can be raised or lowered, and a gripper that can be opened or closed. Each action sub-system generates a 9-element motor vector:

$$\mathbf{v} = \left[v_{lws-}, v_{lws+}, v_{rws-}, v_{rws+}, v_{vert}, v_{horiz}, v_{floor}, v_{open}, v_{close}\right]$$

where $0 \leq v_j \leq 1 \forall v_j \in \mathbf{v}$. Elements 1-4 of **v** denote the backward and forward components of the desired left and right wheel speeds, elements 5-7 indicate alternative positions of the robot arm, and elements 8-9 are instructions to either open or close the gripper.

The condition-action mapping employed by each action sub-system to generate a motor vector and a busy signal value (where needed) are given in Table S1 in pseudocode. Note that for the two 'fixed action patterns', cylinder-pickup and cylinder-deposit, action outputs are determined, in part, by internal clocks, t_{pick} and t_{dep}, that encode elapsed time since the initiation of the behaviour.

The following variables are calculated based on the current sensor input and provided to action sub-systems for use in computing wheel speeds:

$$nws = \begin{cases} 0.07 : ir_{diff} < 30 \\ ir_{diff}/450 : ir_{diff} < 450, \\ 1.0\text{: otherwise} \end{cases}$$

$sws = 0.4 - 4.0 \times 10^{-4}|ir_{tot} - 1200|$, and $fws = 0.4 - 3.5 \times 10^{-4}|ir_{tot} - 1200|$.

The motor output generated by each action sub-system is gated by a shunting inhibition mechanism labelled *SI* in figure S1. As defined in section 3.1 of the main article, the gating signal e_i $(0 \leq e_i \leq 1)$ for the *i*th sub-system is calculated as $e_i = \mathrm{L}(1 - y_i^{snr}/\mathrm{c})$, where $L(a)$ is the piecewise linear function:

$$L(a) = \begin{cases} 0: a < 0 \\ a: 0 \leq a \leq 1. \\ 1: a > 1 \end{cases}$$

Here, c is a constant equal to the value of y_i^{snr} obtained when the basal ganglia model is run to convergence with zero salience input on all channels. In the experiments reported here $\mathrm{c} = 0.169$. The gating signal, e_i, is applied multiplicatively to adjust the gain of the relevant channel. The gated motor outputs are then summed and passed through a further limiter to constrain their values to the range 0 to 1. This gives the aggregate motor vector:

$$\hat{\mathbf{v}} = L(\textstyle\sum_i e_i \mathbf{v}_i).$$

7. The Motor Plant

The motor plant maps the vector $\hat{\mathbf{v}}$ into commands that can be understood by the robot. The wheel motors can be driven forwards are backwards and are controlled by integer valued commands in the range -20 to $+20$. The gripper arm is raised and lowered by a single motor controlled by an integer-valued command. In the reported experiments, the arm was constrained to the range 152 (raised/vertical) to 255 (fully lowered/horizontal). The gripper motor is controlled by a binary signal with a value of 1 for open and 0 for closed. To generate appropriate command signals, elements of the aggregate motor vector, $\hat{\mathbf{v}}$, where converted to command signals as follows (where any fractional values were rounded to the nearest integer):

Wheel speeds: $lws = 15(\hat{v}_{lws+} - \hat{v}_{lws-})$, $rws = 15(\hat{v}_{rws+} - \hat{v}_{rws-})$.

Arm: unless $\hat{v}_{vert} + \hat{v}_{horiz} + \hat{v}_{floor} = 0.0$ then arm_position $= \frac{152\times\hat{v}_{vert} + 227\times\hat{v}_{horiz} + 255\times\hat{v}_{floor}}{\hat{v}_{vert} + \hat{v}_{horiz} + \hat{v}_{floor}}$.

Gripper: unless $\hat{v}_{open} + \hat{v}_{closed} = 0.0$ then $gripper\_position = \begin{cases} 1\text{ (closed)}: \hat{v}_{closed} - \hat{v}_{open} + \hat{v}_{floor} > 0.0 \\ 0\text{ (open): otherwise} \end{cases}$.

Table S1. The condition-action mapping employed by each action sub-system to generate a motor vector and a busy signal value (where needed) at each time-step. Reproduced with permission from [36]. 2006. Elsevier Science and Engineering Journals.

cylinder-seek:		
ir_{tot}≤500	$\mathbf{v}_{seek}$= (0,1.00, 0,1.00, 0,0,0, 0,0)	**// no nearby objects**
		// fast ahead
ir_{tot}>500 & n_{lit} ≥2		**// strong light (nest)**
sd= left:	$\mathbf{v}_{seek}$= (0.27,0, 1.00,0, 0,0,0, 0,0)	// backup, rotating right
sd= right:	$\mathbf{v}_{seek}$= (1.00,0, 0.27,0, 0,0,0, 0,0)	// backup, rotating left
500<ir_{tot}≤1025 & n_{lit} <2	$\mathbf{v}_{seek}$= (0,1.00, 0,1.00, 0,0,0, 0,0)	// **nearby object**
		// fast ahead
1025<ir_{tot}≤2000 & n_{lit} <2		**// possible cylinder**
side= left:	$\mathbf{v}_{seek}$= (0.20,0, 0,0.15, 0,0,0, 0,0)	// rotate toward object
side= right:	$\mathbf{v}_{seek}$= (0,0.15, 0.20,0, 0,0,0, 0,0)	// rotate toward object
ir_{tot}>2000 & n_{lit} <2		**// probable wall**
side= left:	$\mathbf{v}_{seek}$= (0,nws, nws,0, 0,0,0, 0,0)	// rotate away (right)
side= right:	$\mathbf{v}_{seek}$= (nws,0, 0,nws, 0,0,0, 0,0)	// rotate away (left)
wall-seek:		
ir_{tot}≤10	$\mathbf{v}_{wall}$= (0,1.0, 0,1.0, 0,0,0, 0,0)	**// in 'free space',**
		**//** fast ahead
10<ir_{tot}≤500	$\mathbf{v}_{wall}$= (0,0.50, 0,0.50, 0,0,0, 0,0)	**// some contact**
		// slow ahead
ir_{tot}>500		**// near an obstacle**
side= left:	$\mathbf{v}_{wall}$= (0,nws, nws,0, 0,0,0, 0,0)	// rotate right
side= right:	$\mathbf{v}_{wall}$= (nws,0, 0,nws, 0,0,0, 0,0)	// rotate left
wall-follow:		
ir_{tot}≤600		**// well away from wall**
side= left:	$\mathbf{v}_{foll}$= (0,0.20, 0,0.27, 0,0,0, 0,0)	// veer-in sharp left
side= right:	$\mathbf{v}_{foll}$= (0,0.27, 0,0.20, 0,0,0, 0,0)	// veer-in sharp right
if 600<ir_{tot}<1200		**// away from wall**
side= left:	$\mathbf{v}_{foll}$= (0,sws, 0,fws, 0,0,0, 0,0)	// veer-in left
side= right:	$\mathbf{v}_{foll}$= (0,fws, 0,sws, 0,0,0, 0,0)	// veer-in right
1200≤ir_{tot}<2000		**// quite near wall**
side= left:	$\mathbf{v}_{foll}$= (0,fws, 0,sws, 0,0,0, 0,0)	// veer-out gently right
side= right:	$\mathbf{v}_{foll}$= (0,sws, 0,fws, 0,0,0, 0,0)	// veer-out gently left
ir_{tot}>2000		**// very close to wall**
side= left:	$\mathbf{v}_{foll}$= (0,0.15, 0.15,0, 0,0,0, 0,0)	// rotate right
side= right:	$\mathbf{v}_{foll}$= (0.15,0, 0,0.15, 0,0,0, 0,0)	// rotate left
n_{touch}≠1	b_{foll}=0	
n_{touch}=1	b_{foll}=1	
cylinder-pickup:		
0<t_{pick}<0.3	b_{pick}= 0, $\mathbf{v}_{pick}$= (0,0.10, 0,0.10, 0,0,0, 0,0)	// slow approach
0.3≤t_{pick}<1.4	b_{pick}= 1, $\mathbf{v}_{pick}$= (0.20,0, 0.20,0, 0,0,0, 1.0,0)	// backup, open gripper
1.4≤t_{pick}<1.8	b_{pick}= 1, $\mathbf{v}_{pick}$= (0,0, 0,0, 0,0,1.0, 0,0)	// lower arm (floor)
1.8≤t_{pick}<2.8	b_{pick}= 1, $\mathbf{v}_{pick}$= (0,0, 0,0, 0,0,0, 0,1.0)	// close gripper
2.8≤t<3.5	b_{pick}= 1, $\mathbf{v}_{pick}$= (0,0, 0,0, 1.0,0,0, 0,0)	// raise arm (vertical)
3.6≤t_{pick}	b_{pick}= 0, $\mathbf{v}_{pick}$= (0,0, 0,0, 0,0,0, 0,0), t= 0.0	// idle
cylinder-deposit:		
0<t_{dep}<0.8	b_{dep}= 1, $\mathbf{v}_{dep}$= (0,0, 0,0, 0,1.0,0, 0,0)	// lower arm (horizontal)
0.8≤t_{dep}<1.6	b_{dep}= 1, $\mathbf{v}_{dep}$= (0,0, 0,0, 0,0,0, 1.0,0)	// release cylinder
1.6≤t_{dep}<2.4	b_{dep}= 1, $\mathbf{v}_{dep}$= (0,0, 0,0, 1.0,0,0, 0,0)	// raise arm (vertical)
2.4≤t_{dep}	b_{dep}= 0, $\mathbf{v}_{dep}$= (0,0, 0,0, 0,0,0, 0,0), t= 0.0	// idle
